# Genomic alterations of ERBB receptors in cancer: clinical implications

**DOI:** 10.18632/oncotarget.22825

**Published:** 2017-11-30

**Authors:** Rosalin Mishra, Ariella B. Hanker, Joan T. Garrett

**Affiliations:** ^1^ Division of Pharmaceutical Sciences, James L. Winkle College of Pharmacy, University of Cincinnati, Cincinnati, Ohio, U.S.A; ^2^ Department of Medicine, Breast Cancer Program, Vanderbilt-Ingram Cancer Center, Vanderbilt University Medical Center, Nashville, Tennessee, U.S.A

**Keywords:** EGFR, HER2, HER3, HER4, mutation

## Abstract

The ERBB family of receptor tyrosine kinases has been implicated in carcinogenesis for over three decades with rigorous attention to EGFR and HER2. ERBB receptors, consisting of EGFR, HER2, HER3, and HER4 are part of a complicated signaling network that activates downstream signaling pathways including PI3K/AKT, Ras/Raf/MAPK, JAK/STAT and PKC. It is well established that EGFR is amplified and/or mutated in gliomas and non-small-cell lung carcinoma while HER2 is amplified and/or over-expressed in breast, gastric, ovarian, non-small cell lung carcinoma, and several other tumor types. With the advent of next generation sequencing and large scale efforts to explore the entire spectrum of genomic alterations involved in human cancer progression, it is now appreciated that somatic ERBB receptor mutations occur at relatively low frequencies across multiple tumor types. Some of these mutations may represent oncogenic driver events; clinical studies are underway to determine whether tumors harboring these alterations respond to small molecule EGFR/HER2 inhibitors. Recent evidence suggests that some somatic ERBB receptor mutations render resistance to FDA-approved EGFR and HER2 inhibitors. In this review, we focus on the landscape of genomic alterations of EGFR, HER2, HER3 and HER4 in cancer and the clinical implications for patients harboring these alterations.

## INTRODUCTION

The ERBB family of receptor tyrosine kinases (RTKs), consisting of EGFR (also known as ERBB1, HER1), HER2 (ERBB2, neu), HER3 (ERBB3) and HER4 (ERBB4), were first implicated in cancer in the beginning of the 1980s when it was discovered that EGFR had close sequence homology to avian erythroblastosis tumor virus (AEV) [1, 2]. HER2/neu was first identified in rat carcinogen-induced tumors with a transmembrane domain mutation, V664E, that made its tyrosine kinase constitutively active [3]. The V664E mutation in HER2 supports receptor dimerization and greater tyrosine kinase activity [4]. The HER2^V664E^ mutation has not yet been found in human tumors. HER3 and HER4 were subsequently identified due to their sequence homologies to EGFR [5-7]. Each member of the ERBB family is composed of an extracellular ligand-binding domain, a single transmembrane domain and an intracellular domain which includes the tyrosine kinase (TK) domain. Signaling in the EGFR family is typically initiated when ligands bind the ectodomain, causing conformational changes that allows for homo- or heterodimerization with other ERBB family members. Dimerization activates cytoplasmic catalytic activity resulting in trans- and autophosphorylation of tyrosine residues in the cytoplasmic tails. These tyrosine residues serve as docking sites for several adapator proteins which initiate multiple signaling cascades, ultimately resulting in deregulated cell proliferation, cell survival, angiogenesis, and metastasis.

**Figure 1:**
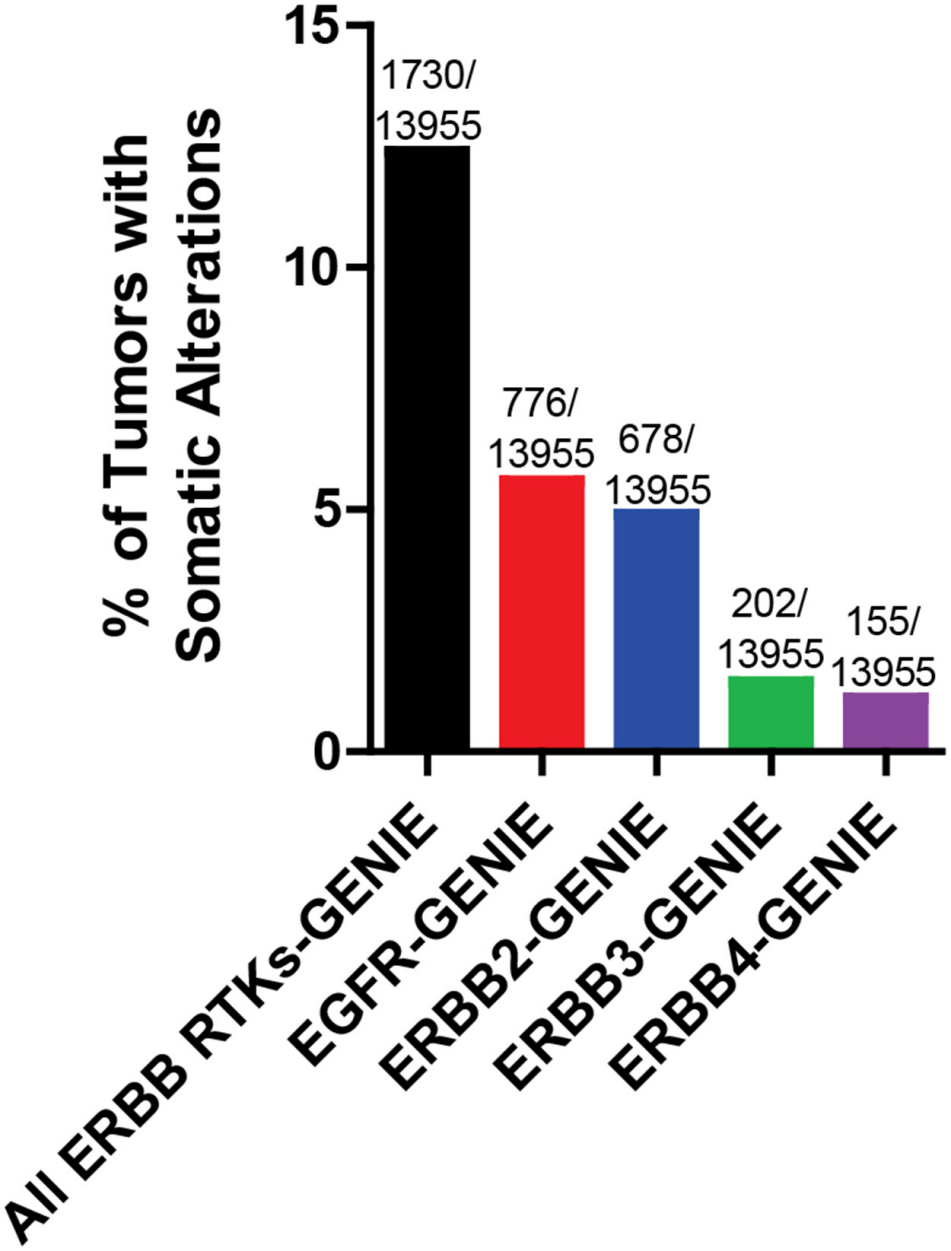
Frequency of somatic alterations of ERBB receptors in cancer The Project GENIE dataset was analyzed for frequency of *EGFR, ERBB2, ERBB3*, and *ERBB4* copy number amplification and putative driver mutations across all cancer types (N=13955 tumors with copy number and mutation data for all 4 genes). Some tumors harbored multiple alterations. Putative driver mutations are defined as: cancer hotspot or OncoKB driver annotation (defined by cBioPortal.org) or number >5 in cBioPortal or COSMIC datasets.

The advent of next generation sequencing technology has allowed for many large scale projects exploring whole genome or exome analysis of tumors including TCGA (The Cancer Genome Atlas) and ICGC (International Cancer Genome Consortium) [8]. Figure 1 indicates the frequency of ERBB receptor copy number amplification and putative driver mutations across all cancer types from the GENIE data set [9]. A putative driver mutations is defined as frequency>5 in cBioPortal or COSMIC databases, or a HotSpot or OncoKB driver annotation in cBioPortal. In accordance with an abundance of literature indicating the oncogenic role of *EGFR* and *ERBB2*, these two family members each have a greater than three-fold higher incidence of somatic alterations compared to *ERBB3* or *ERBB4*. Nevertheless, the frequency rate of somatic alterations of EGFR account for only 5.6% of all cancer types, with other ERBB family members having a lower rate of somatic alterations within the GENIE dataset [9]. However, we note that this may be an underestimate, as many of the sequencing assays used in project GENIE fail to detect gene rearrangements and large deletions, such as the EGFR type III variant, frequently found in glioblastoma. Collectively, greater than 12% of all cancers examined in the GENIE data set harbor somatic alterations in one or more members of the ERBB family. Further efforts are underway to distinguish between ERBB receptor mutations that drive cancer progression versus passenger mutations. Passenger mutations are not thought to contribute to cancer growth; rather, they simply accrue during the course of tumor development as a result of genomic instability. In this review, we discuss recent advances in our understanding of genomic alterations of the ERBB family members in cancer and efforts to target these alterations.

## EGFR MUTATIONS IN NON-SMALL CELL LUNG CANCER

**Figure 2:**
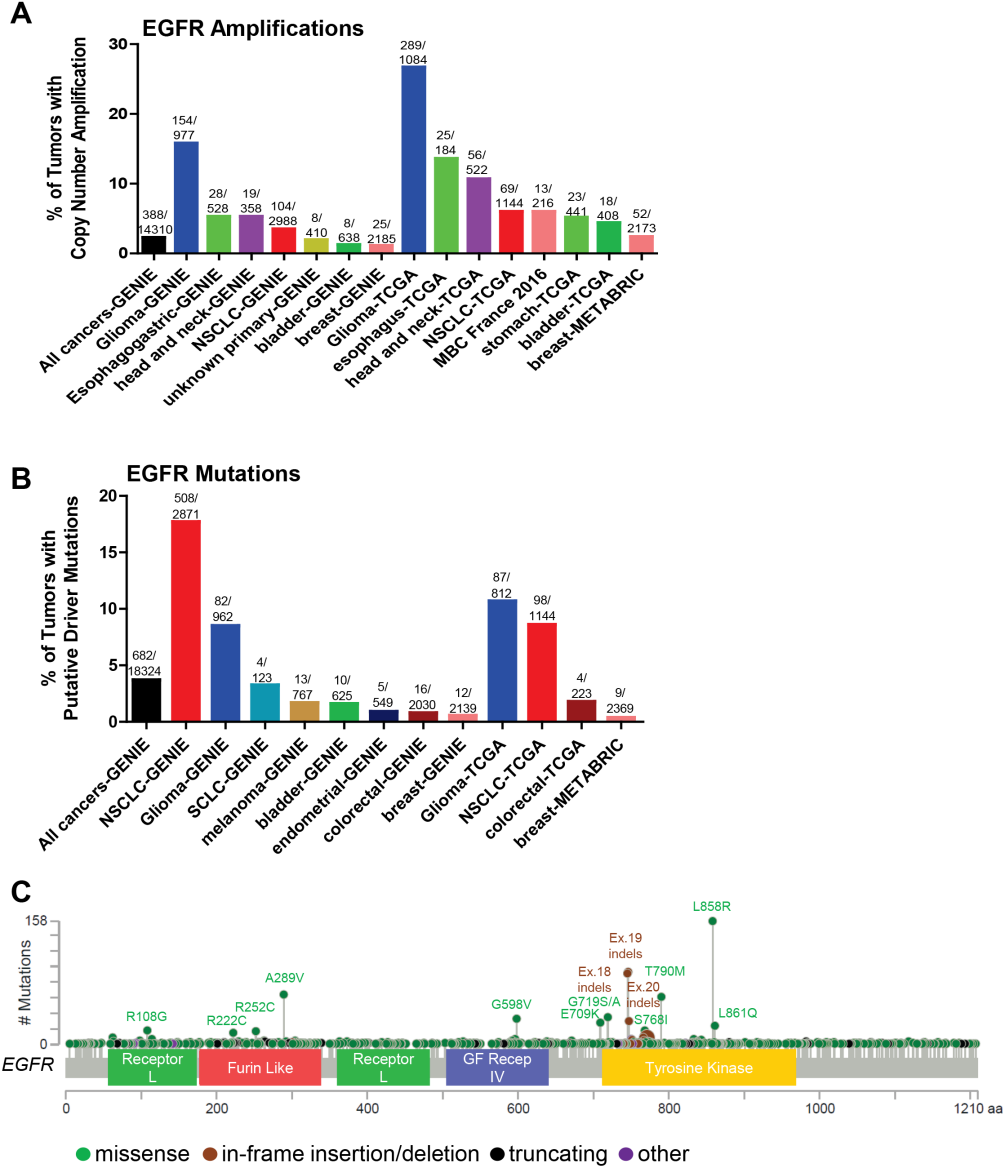
Somatic alterations of *EGFR* in cancer **(A, B)** Frequency of *EGFR* copy number amplifications (A) or putative driver mutations (B) in selected cBioPortal and GENIE datasets. **(C)** Distribution of somatic variants within *EGFR* across its domain-annotated protein structure in all cBioPortal studies. NSCLC, non-small cell lung cancer; SCLC, small cell lung cancer; GF Recep IV, Growth Factor Receptor IV domain.

The selective response of non-small cell lung cancer (NSCLC) patients to EGFR tyrosine kinase inhibitors (TKIs) gefitinib and erlotinib allowed for the identification of oncogenic EGFR mutations [10–13]. Many EGFR activating mutations are found in the catalytic kinase domain (exons 18-24) including small in-frame deletions found at amino acids 747-750 of exon 19 and the L858R mutation in exon 21, the most frequent EGFR mutation (Figure 2C). These activating mutations are clustered around the ATP-binding pocket of the enzyme [14] and display up to a 50-fold acceleration in catalysis by disrupting autoinhibitory interactions [15]. Increased kinase activity of EGFR results in pro-survival and anti-apoptotic signals via activation of downstream targets including PI3K-AKT, ERK and STAT. Thus, these mutations represent classic cases of oncogene addiction [16]. As such, the efficacy of the first-line EGFR inhibitors gefitinib and erlotinib over cytotoxic chemotherapy in patients with EGFR-mutant NCSLC has been well-established [17].

Despite the successes of EGFR targeting agents in patients with activating catalytic kinase domain EGFR mutations compared to chemotherapy, patients invariably progress within several years of treatment. The first identified mechanism of acquired resistance to EGFR TKIs was the EGFR T790M mutation [18, 19]. The T790M mutation structurally corresponds to the mutated gatekeeper residue T315I in BCR-ABL, T670I in c-KIT and T674I in PDGFRα [20]. The EGFR T790M mutation has increased affinity to ATP, resulting in decreased sensitivity to ATP-competitive reversible inhibitors [21]. Notably, the T790M mutation is one of the most frequently found mutations in EGFR (Figure 2C), with the caveat that 57 of 63 of these T790M EGFR mutant tumors come from the MSK-IMPACT cohort [22]. While the T790M mutation is very rare in primary untreated tumors [23], it has a much higher frequency in the project GENIE dataset [9], which includes tumors that have relapsed following treatment with EGFR TKIs. However, there is evidence showing that the T790M mutation can be found in primary, untreated tumors, and also in germline cells in families with inherited lung cancer [24, 25]. Thus, the detection of the EGFR T790M mutation in tumors could indicate a dependence on EGFR due to increased ATP-binding to EGFR. Afatinib is a second-generation irreversible, covalently-bound inhibitor of EGFR that has more recently been approved to treat NSCLCs harboring EGFR activating mutations [26] but still may have decreased sensitivity to tumors harboring T790M mutations [27]. Other classes of drugs targeting EGFR-mutant tumors are in various stages of development, including mutant-selective irreversible inhibitors, such as osimertinib (AZD9291), based on a pyrimidine scaffold that forms a covalent bond with Cys797 at the edge of the ATP binding pocket [28]. In 2015, osimertinib received accelerated FDA approval for NSCLC patients carrying the T790M mutation, and had recently shown promising efficacy as a first-line treatment for EGFR-mutant NSCLC [29]. However, as with first- and second-generation EGFR TKIs, resistance to osimertinib ultimately develops. Potential mechanisms of resistance include loss of the T790M mutation [30], mutations in the RAS pathway [31], amplification of *ERBB2* or *MET* [29, 30], and the EGFR C797S mutation, altering the amino acid that binds osimertinib [29, 32, 33]. The latter mutation was found in 40% of a small cohort of NSCLC patients following acquired resistance to osimertinib [33]. EA1045 is an inhibitor that binds the allosteric pocket of EGFR rather than the ATP binding pocket, and is selective for drug-resistant EGFR mutants [34]. The combination of EA1045 and cetuximab blocked the growth of mouse models of lung cancer harboring EGFR T790M and C797S.

Other recurrent EGFR mutations in NSCLC include the exon 18 mutations E709K and G719A/C/D/S, the exon 20 S768I missense mutation, and exon 20 insertions (Table 1 and Figure 2C). While there is evidence that the missense mutations respond to second-generation EGFR inhibitors such as afatinib, the Exon 20 insertions are thought to be less sensitive [35]. Mutant-specific EGFR inhibitors with preclinical activity against the exon 20 insertion mutations, such as AP32788 and EGF816, are now in clinical development [36, 37].

**Table 1:** Selected ERBB family mutations found in patients

ERBB mutation	Principle tumor type (cBioPortal)	Drug sensitivity	Drug insensitivity	Acquired resistance
**EGFR R108G/K**	Glioma	preclinical: erlotinib [143]		
**EGFR A289D/I/N/T/V**	Glioma	preclinical: erlotinib [143]		
**EGFR E709A/K/Q**	NSCLC	preclinical: afatinib, neratinib [144]		
**EGFR exon 18 insertions/deletions**	NSCLC	preclinical, clinical: afatinib, neratinib [144]		
**EGFR G719A/C/D/S**	NSCLC	clinical: erlotinib, gefitinib, afatinib [145], [35]		
**EGFR exon 19 insertions/deletions**	NSCLC	clinical: erlotinib, gefitinib, afatinib [146]		
**EGFR exon 20 insertions/deletions**	NSCLC	preclinical: EGF816, AP32788 [36, 37]	clinical: afatinib, gefitinib, erlotinib [147],[148],[35]	
**EGFR S768G/I/T**	NSCLC	clinical: afatinib, gefitinib, erlotinib [149], [35]		
**EGFR T790M**	NSCLC	clinical: osimertinib [28]; preclinical: EA1045 and cetuximab [34]	clinical: afatinib [26, 27]	clinical: gefitinib, erlotinib, afatinib [18, 19]
**EGFR C797S/Y**	NSCLC	preclinical: EA1045 and cetuximab [34]		clinical: osimertinib [32, 33]
**EGFR L858R**	NSCLC	clinical: gefitinib, erlotinib, afatinib [10],[11]		
**EGFR L861Q/R**	NSCLC, lung squamous	clinical: gefitinib, erlotinib, afatinib [145], [35]		
**ERBB2 D277G/H/V/Y**	bladder	preclinical: lapatinib, afatinib [80]		
**ERBB2 S310F/Y**	bladder, breast, esophagogastric, colorectal, lung, cervical	preclinical: trastuzumab, lapatinib, neratinib, afatinib [77, 79, 80, 90]; clinical: neratinib [103, 105]	preclinical: cetuximab, panitumumab [79]	
**ERBB2 R678Q**	esophagogastric, colorectal, bladder	preclinical: lapatinib, afatinib, neratinib [77, 80]		
**ERBB2 L755S**	breast, bladder, colorectal	preclinical: neratinib, afatinib [77, 112]; clinical: neratinib [98, 103, 105]	preclinical: trastuzumab, lapatinib, cetuximab, panitumumab [77, 79, 85, 112]	preclinical: lapatinib [112]
**ERBB2 D769H/Y**	breast, bladder, esophagogastric, colorectal	preclinical: trastuzumab, lapatinib, neratinib [77]; clinical: neratinib [103]		
**ERBB2 exon 20 insertions/deletions**	NSCLC	preclinical: lapatinib, afatinib, neratinib, AP32788 [36, 73, 77, 82, 84, 93]; clinical: trastuzumab, afatinib, neratinib, dacomitinib [96, 100-103, 105] [150], [97]	preclinical: erlotinib, gefitinib [73, 82, 93]	clinical: osimertinib [29]
**ERBB2 V777L**	breast, colorectal, esophagogastric	preclinical: trastuzumab, lapatinib, neratinib [77, 79, 85] clinical: neratinib [91]	preclinical: cetuximab, panitumumab [79]	
**ERBB2 T798I/M**	breast	preclinical: afatinib [91]	preclinical: trastuzumab, lapatinib, neratinib [85, 91, 111]	clinical: neratinib [91]
**ERBB2 V842I**	colorectal, breast, esophagogastric, endometrial	preclinical: trastuzumab, lapatinib, neratinib [77, 79]; clinical: neratinib [103]	preclinical: cetuximab, panitumumab [79]	
**ERBB2 L869R**	breast	preclinical: neratinib, afatinib [91]; clinical: neratinib [92, 103, 105]	preclinical: lapatinib [91]	
**ERBB3 V855A**	NSCLC	preclinical: pertuzumab and afatinib [128]		
**ERBB3 mutations**	multiple tumor types		clinical: neratinib [105]	
**ERBB4 KD mutations**	melanoma, esophagogastric, colorectal	preclinical: lapatinib [132]		

## THERAPEUTIC CHALLENGES IN GLIOMA WITH EGFR GENETIC ALTERATIONS

Grade IV glioblastoma multiform (GBM) is the most common and aggressive cancer originating in the central nervous system (CNS), exhibiting a high frequency of recurrence and dismal prognosis due to the invasive nature of the tumor. The standard of care for GBM patients is surgical resection followed by radiation plus the chemotherapeutic temozolomide, with a median overall survival of 15 months from diagnosis [38]. GBM is frequently associated with molecular changes in EGFR (Figure 2). The most common and best-studied EGFR alteration in glioblastomas is the EGFR type III variant (EGFRvIII), a constitutively active genomic deletion variant lacking exons 2 to 7 of the EGFR gene, usually occurring in EGFR-amplified tumors [39–41]. EGFRvIII lacks domains I and II of the extracellular region of wild-type (WT) EGFR. Lacking the domain II loop, EGFRvIII is thought to avoid formation of the tethered, inactive conformation, causing a shift in the equilibrium to the open, active conformation [42]. Large-scale genomic studies reported that EGFR is a key driver of GBM, defining a subtype of GBM [43]. Genetic alterations including mutations, rearrangements, alternative splicing and focal amplifications occurred in 57% of primary GBMs [44]. Thus, EGFR represents a prime therapeutic target for glioblastoma. Unfortunately, the use of the EGFR tyrosine kinase inhibitors erlotinib [45, 46], gefitinib [47, 48], afatinib [49], or lapatinib [50], either alone or in combination with other agents, has resulted in disappointing results in the clinic. In addition to drug delivery concerns in glioblastoma, there are several important caveats to consider. The disappointing exploration of EGFR as a target in glioblastoma includes well-documented intra-tumoral heterogeneity of EGFR and amplification of other RTKs that could bypass EGFR inhibition [51–54]. One mechanism for *de novo* resistance in glioblastoma to EGFR inhibitors is the ability of these cancers to reversibly up-regulate or suppress mutant EGFR expression, resulting in distinct cellular phenotypes to reach an optimal equilibrium for growth [55]. Although one of the most characteristic features of glioblastoma is alterations in EGFR, therapeutically targeting EGFR is currently not efficacious likely due to heterogeneity of EGFR signaling networks and redundant alternative signaling pathway activation [56].

Novel strategies to target EGFR in GBM are currently being explored. One area of interest for patients harboring EGFR amplifications are antibody-drug conjugates (ADCs) [57]. ADCs enable therapeutic delivery of cytotoxic agents specifically to tumor cells by linkage to an antibody targeting a protein that is expressed more highly on tumor cells than normal cells. The ADC ABT-414 is comprised of an EGFR antibody linked to monomethyl auristatin F (MMAF), an inhibitor of tubulin assembly. ABT-806, the parental antibody of ABT-414, was found to accumulate specifically in the tumor in both mouse models and in patients with glioma [58, 59] and showed impressive antitumor activity in GBM xenografts harboring EGFR amplification or EGFRvIII [60]. Phase I trials found that ABT-414 demonstrated an acceptable safety profile, although ocular toxicity was very common [61], and several partial responses were observed. ABT-414 is currently in phase II studies as monotherapy or in combination with temozolomide. Since EGFRvIII is specific to tumor cells, it has also been an attractive target for immunotherapeutic approaches [62]. These include rindopepimut (CDX-110-KLH), a 14 amino acid peptide vaccine corresponding to the fusion junction of EGFRvIII [63]. This peptide was then used as the basis for a dendritic cell (DC) vaccine. In preclinical studies, DCs pulsed with CDX-110-KLH caused prolonged immunity and a significant elongation of survival in mouse models [64]. However, this vaccine failed to prolong overall survival in a phase III trial [65]. Due to the substantial intracellular heterogeneity in GBM, combination therapies may be needed to block the growth of all tumor cells.

## HER2 ALTERATIONS IN CANCER

**Figure 3:**
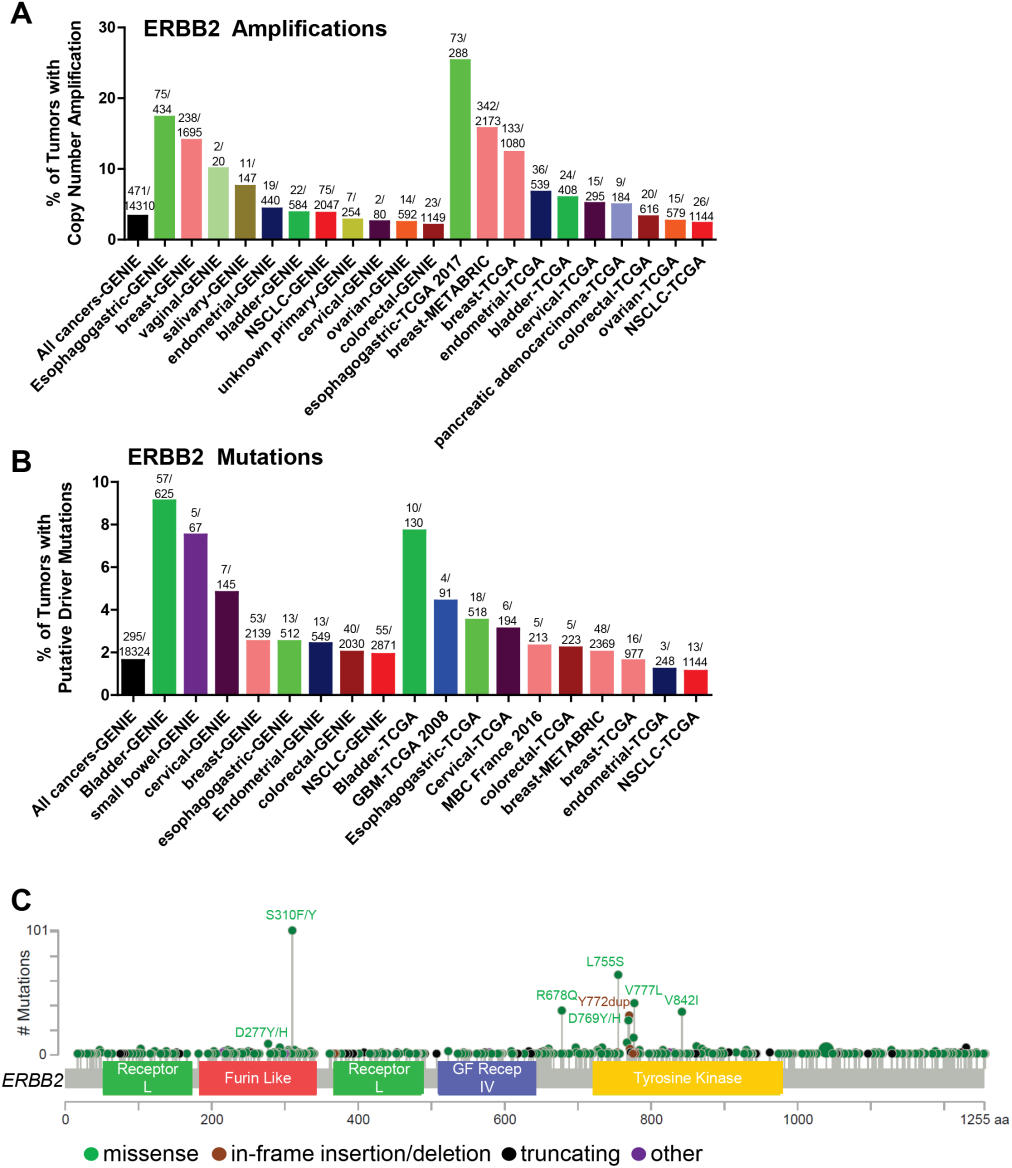
Somatic alterations of *ERBB2* in cancer **(A, B)** Frequency of *ERBB2* copy number amplifications (A) or putative driver mutations (B) in selected cBioPortal and GENIE datasets. **(C)** Distribution of somatic variants within *ERBB2* across its domain-annotated protein structure in all cBioPortal studies. NSCLC, non-small cell lung cancer; GBM, glioblastoma multiform; MBC, metastatic breast cancer; GF Recep IV, Growth Factor Receptor IV domain.

HER2 has long been known to be amplified and overexpressed in breast [66], gastric [67], and bladder cancers [68] and large scale copy number analysis confirms that HER2 is amplified most frequently in gastric followed by breast cancer (Figure 3A). As such, several decades of drug discovery efforts have resulted in five HER2 inhibitors that are currently FDA-approved to treat HER2-amplified/overexpressing breast cancers. These include the monoclonal antibodies trastuzumab and pertuzumab, the ADC trastuzumab emtansine, and the EGFR/HER2 TKIs lapatinib and neratinib. These drugs, in combination with chemotherapy, have significantly improved outcomes for HER2-amplified breast cancer patients, particularly in the adjuvant setting. However, resistance to HER2 inhibitors in metastatic cancers remains a problem. Potential mechanisms of resistance include activation of downstream signaling pathways, such as the PI3K/AKT pathway, HER2 truncations (p95 HER2) and splice variants, upregulation of ERBB ligands, and activation of other RTKs [69].

Unlike breast cancer, only trastuzumab is approved to treat HER2-amplified gastric cancer. Trastuzumab emtansine and lapatinib failed to prolong survival over standard treatment. The combination of pertuzumab, trastuzumab, and chemotherapy is currently being evaluated in the phase III JACOB trial for HER2-positive gastric cancers [70]. Trastuzumab and lapatinib and trastuzumab emtansine are currently being investigated in the clinic for other types of cancer with HER2 amplifications, including lung and colorectal cancers.

Somatic mutations in the kinase domain (KD) of the *ERBB2* gene (primarily exon 20 insertions) were first reported in a low frequency of lung cancers [71, 72] and were subsequently shown to increase HER2 protein activation [73]. More recently, HER2 mutations have been found in a variety of cancer types [74, 75] (Figure 3B), including breast [76–78], colorectal [79], and bladder cancers [80]. HER2 somatic mutations correlate with poor survival in HER2-negative (non-amplified) breast cancer [81]. Nearly 2% of all cancers harbor hotspot or putative activating mutations in *ERBB2* (Figure 3B), suggesting that these cancers may be sensitive to HER2-targeted therapies. The most frequent HER2 mutations are found in the extracellular domain ECD (primarily S310F/Y) and the KD (exon 20 insertions/deletions, L755S, V777L, V842I; Figure 3C). The S310F/Y mutation is most frequently found in bladder cancer, whereas the KD mutations L755S and V777L occur frequently in breast cancer, and V842I is most commonly found in colorectal cancer. The majority of HER2 mutations in lung cancer are the exon 20 insertions/deletions, the most prevalent being the Y772_A775 duplication (also known as the A775_G776 YVMA insertion). The exon 20 insertions were the first HER2 mutations to be extensively characterized [73, 82]. The exon 20 insertions are similar to those found in EGFR [71] and are thought to induce a conformational change of the autoinhibitory αC-β4 loop in the kinase domain, narrowing the ATP-binding cleft and leading to enhanced kinase activity [83]. HER2^YVMA^ was further shown to increase phosphorylation of HER2, EGFR, and downstream signal transducers including AKT and ERK, transform bronchial and mammary epithelial cells, and promote tumor formation in nude mice [73]. Inducible expression of HER2^YVMA^ in the mouse lung epithelium promoted development of adenosquamous lung tumors that were sensitive to HER2 kinase inhibition [84].

Bose et al. characterized the HER2 activating mutations G309A, D769H/Y, V777L, P780ins, and V842I. These mutations promoted the HER2 kinase activity, phosphorylation of HER2, EGFR, HER3, and ERK, and the transformation of mammary epithelial cells [77]. Similarly, the L755S/P, V777L, and T862A mutations promoted colony formation in NMuMg mouse mammary epithelial cells [85], and the L755S, V777L, V842I, and S310F mutations were shown to transform colonic epithelial cells [79]. Of note, the V777L mutation is homologous to EGFR V769L, a rare mutation associated with NSCLCs (Table 2), and ALK F1174L, a known activating mutation in neuroblastoma [77, 86–89]. The V777 residue abuts the conserved DFG motif involved in tyrosine kinase activity [77].

**Table 2:** Sequence homology of missense mutations found in ERBB family members

EGFR	ERBB2	ERBB3	ERBB4
R108G/K (glioma)	R103Q (bladder)	ND	R106C/H (multiple)
ND	S310F/Y (multiple)	ND	S303F/Y (multiple)
S768I (NSCLC)	G776V/S (multiple)	ND	ND
V769L (NSCLC)	V777A/L/M (multiple)	ND	ND
T790M (NSCLC)	T798I (breast)	ND	ND
L858R (NSCLC)	ND	V855A (NSCLC)	ND
L861R/Q (NSCLC; lung squamous)	L869R/Q (breast)	ND	ND
ND	V842I (multiple)	ND	V840I (multiple)

ND (no data): mutation not reported/not found in cBioPortal or GENIE.

Greulich et al. reported that the HER2 ECD mutations G309E, S310F, and S310Y, in addition to several rare ECD mutants identified in glioblastoma, increased colony formation in NIH 3T3 cells [90]. While the rare G309E mutation promoted covalent homo-dimerization mediated by intermolecular disulfide bond formation, HER2^S310F^ functioned more similarly to the kinase domain mutations, and increased c-terminal tail phosphorylation. Several studies reported that many non-“hotspot” HER2 variants of unknown significance (VUS) do not appear to activate HER2 kinase activity or signaling [77, 80, 90]. However, we recently reported that the relatively rare HER2^L869R^ mutation increased HER2-mediated signaling and growth of MCF10A mammary epithelial cells; in addition, a breast cancer patient with this mutation showed an excellent response to neratinib [91]. Therefore, more studies are needed to characterize the large number of HER2 VUS appearing in databases such as cBioPortal and GENIE in order to determine if they are gain-of-function driver mutations or neutral passenger mutations.

Recent evidence suggests that HER2 mutants, when expressed at endogenous levels, demonstrate weak oncogenic properties [92] and require additional cooperating mutations to transform cancer cells. Such cooperating alterations may include co-occurring mutations in *PIK3CA* [92] and *ERBB3* [91]. In addition, low-level copy number gain of *ERBB2* is frequently observed in HER2-mutant tumors (www.cbioportal.org); therefore, elevated expression of the HER2 mutants may contribute to their oncogenic function.

## THERAPEUTIC TARGETING OF MUTANT HER2

There has been considerable interest in determining whether the growing number of anti-HER2 therapies initially developed to treat HER2-amplified breast cancer could also block cancers harboring HER2 mutations. A number of studies have examined whether cells engineered to express various HER2 mutants are sensitive to HER2 TKIs and monoclonal antibodies. Moderate sensitivity to trastuzumab was observed in MCF10A cells expressing various HER2 mutants [73, 77]. Some mutants, including S310F/Y and V777L, were sensitive to lapatinib [77, 85, 90], whereas others, such as L755S, L869R, and exon 20 insertions/deletions, displayed lapatinib resistance *in vitro* [77, 85, 91]. Most mutants tested were sensitive to irreversible EGFR/HER2 inhibitors such as afatinib and neratinib [73, 77, 90, 93]. Perera et al. found that transgenic mouse tumors driven by the HER2 YVMA insertion were somewhat sensitive to afatinib, but the combination of afatinib with the mTOR inhibitor rapamycin was more efficacious [84].

Several studies have examined the sensitivity of cells lines harboring naturally occurring HER2 mutations to HER2 inhibitors. Early studies indicated that the H1781 lung cancer cell line, harboring the HER2 G776_VC insertion, is sensitive to combined treatment of lapatinib and trastuzumab, the pan-HER inhibitor CI-1033, and neratinib [73, 82]. Urinary bladder cancer (UBC) cell lines harboring the HER2 mutations S653C, R678Q, and S310F overall were more sensitive to lapatinib than HER2 WT cell lines [80]. Bose and colleagues examined whether colorectal cancer patient-derived xenografts (PDXs) harboring HER2 mutations responded effectively to HER2 inhibitors [79]. PDXs harboring HER2^S310Y^ or HER2^L866M^ were resistant to the EGFR monoclonal antibodies cetuximab and panitumumab, but were sensitive to neratinib. The HER2^S310Y^-expressing PDX was partially sensitive to trastuzumab or lapatinib as single agents. In both xenografts, the combination of neratinib and trastuzumab led to more complete inhibition of tumor growth, suggesting that this combination should be explored further.

In addition to preclinical studies, there are several case studies reporting that individuals with HER2-mutant metastatic lung or breast cancer respond to trastuzumab, trastuzumab + pertuzumab, or neratinib [94–97]. Due to the robust preclinical data, there is significant interest in using the irreversible EGFR/HER2 inhibitors neratinib and afatinib to treat HER2-mutant cancers. Responses to these agents have been documented in individuals with HER2-mutant lung and breast cancers [91, 96, 98–101]. Early results of clinical trials testing HER2 TKIs in HER2-mutant cancers are promising. In 30 patients with NSCLC harboring HER2 exon 20 mutations (primarily insertions) treated with the irreversible EGFR/HER2/HER4 inhibitor dacomitinib, 3 partial responses were seen [102]. The MutHER and SUMMIT trials tested the efficacy of neratinib in HER2-non-amplified, HER2-mutant metastatic breast cancer. Clinical benefit rates of 31% and 41.7% to neratinib monotherapy were observed in the MutHER and SUMMIT trials, respectively [103, 104]. The clinical benefit rate increased to 58.3% in patients treated with neratinib in combination with the ER antagonist fulvestrant in the SUMMIT trial. Complete responses were observed in patients with V777L, L755S, and S310F missense mutations and the GSP and YVMA insertions. In the MutHER trial, no responses were seen in the uncharacterized VUS S609F and P802S [103]. The SUMMIT trial also tested the efficacy of neratinib monotherapy in other cancer types with HER2 mutations (n=125 patients representing 21 cancer types and 30 HER2 mutations) [105]. The greatest clinical activity was seen in breast, cervix, and biliary cancers, and with tumors harboring kinase domain missense mutations. These results strongly suggest that some HER2 mutations are true “driver” mutations in these cancers. No clinical benefit was observed in HER2-mutant colorectal cancers, regardless of mutation type, suggesting that overall response rates may be influenced by both tissue type and mutation type.

As with most targeted therapies in advanced cancers, acquired resistance to neratinib monotherapy is expected. An acquired HER2^T798I^ “gatekeeper” mutation, homologous to the EGFR^T790M^ mutation in EGFR inhibitor-resistant lung cancer (Table 2), was recently identified in a HER2-mutant breast cancer patient following progression on neratinib [91]. Structural modeling of the HER2^T798I^ mutant suggested that the increased bulk of the isoleucine in place of the threonine sterically blocked neratinib binding. Afatinib, but not neratinib, blocked the growth of HER2^T798I^-expressing cells, perhaps because afatinib is smaller than neratinib and may not be occluded from the binding pocket. Interestingly, the same mutation was also found in another HER2-mutant breast cancer patient following progression on neratinib [103]. Several other acquired HER2 mutations were also found in the circulating tumor DNA (ctDNA) of patients following progression on neratinib, including R678Q, V697L, T862A, and I767M; more than one acquired HER2 mutation was found in the same patient. These studies strongly suggest that these tumors were “addicted” to the initial driver alteration(s) in HER2, and required re-activation of HER2 for continued growth. Recently, the HER2^C805S^ mutation, homologous to the drug-resistant EGFR^C797S^ mutation, was found to promote resistance to irreversible HER2 TKIs in HER2-mutant cells *in vitro* [106], but this mutation has not yet been found in patients. Other potential mechanisms of acquired resistance to neratinib include *PIK3CA* mutations and amplification of the mutant *ERBB2* allele [96, 107].

Although activating HER2 mutations are found in ∼2-6% of HER2-amplified breast cancers (www.cbioportal.org; [78, 108]) whether they promote resistance to HER2-targeted therapy in HER2-amplified breast cancer has not yet been clearly established. Boulbes *et al.* sequenced the kinase domains of *EGFR, ERBB2*, and *ERBB4* in 76 primary HER2+ breast cancers and found 6 mutations in *EGFR*, 3 in *ERBB2*, and 3 in *ERBB4*. None of the patients with ERBB mutations responded to trastuzumab, whereas 32% of patients with ERBB-WT cancers achieved partial responses. They further showed that the novel HER2^L726F^ mutation detected in a patient reduced lapatinib efficacy in HER2-amplified BT474 breast cancer cells [109]. Larger studies are needed to confirm whether ERBB family mutations are associated with *de novo* resistance to HER2 inhibitors. In another study, acquired HER2 mutations were identified in 5/16 metastatic breast cancer samples treated with adjuvant trastuzumab. The mutations were not detected in matched primary samples. Three of these were the known lapatinib-resistant L755S mutation, whereas two were the novel K753E mutation. The authors further showed that HER2^K753E^ expression promoted resistance to lapatinib and trastuzumab [110]. Similarly, recent preclinical studies have identified HER2 L755S and T798M as mutations that promote resistance to lapatinib or trastuzumab [111, 112]. In addition, HER2^T798M^ was shown to be insensitive to neratinib in BT474 cells [91]. Afatinib retained its ability to block HER2 in these cells, while cells expressing HER2^L755S^ retained sensitivity to afatinib and neratinib [112], Therefore, HER2-positive patients progressing on anti-HER2 therapy should be profiled for acquired drug-resistant HER2 alterations.

Amplification of HER2 is a well-established mechanism of acquired resistance to EGFR TKIs in NSCLC [113, 114]. However, whether HER2 mutations also promote acquired resistance to EGFR TKIs is not yet known. Interestingly, in primary lung tumors, HER2 and EGFR activating mutations are mutually exclusive [115], suggesting that they have overlapping roles in oncogenesis. A HER2 exon 20 insertion was recently found in an EGFR-mutant lung tumor with acquired resistance to osimertinib; no additional cancer-associated mutations were found in this tumor [29]. Future studies should investigate whether HER2 mutations directly promote resistance to osimertinib and other EGFR TKIs.

## HER3 MUTATIONS AND AMPLIFICATIONS

**Figure 4:**
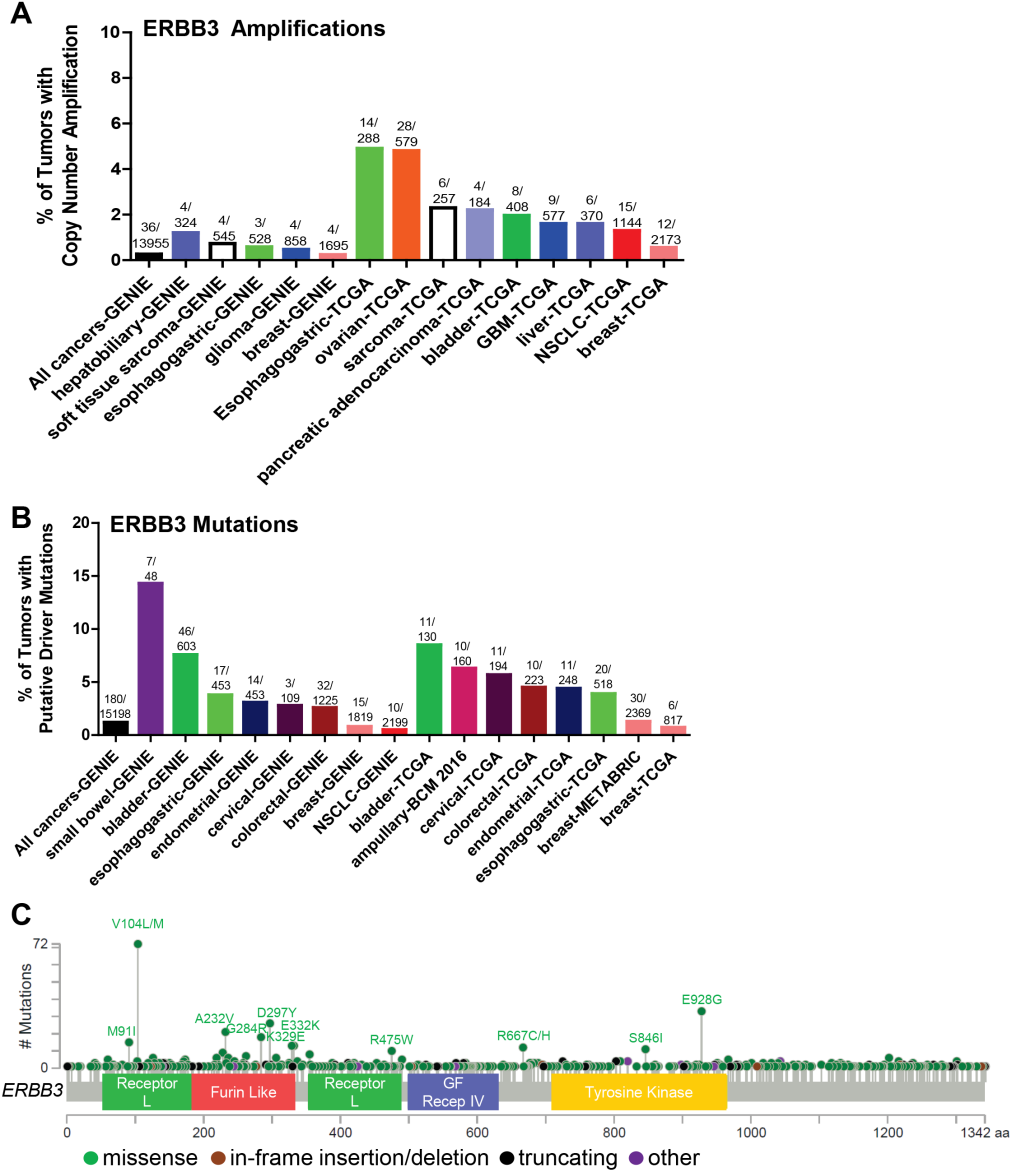
Somatic alterations of *ERBB3* in cancer **(A, B)** Frequency of *ERBB3* copy number amplifications (A) or putative driver mutations (B) in selected cBioPortal and GENIE datasets. **(C)** Distribution of somatic variants within *ERBB3* across its domain-annotated protein structure in all cBioPortal studies. NSCLC, non-small cell lung cancer; GBM, glioblastoma multiforme; GF Recep IV, Growth Factor Receptor IV domain.

Although somatic mutations and alterations associated with EGFR and HER2 have been studied more rigorously due to their well-established roles as oncogenes, HER3 (*ERBB3*) mutations have been in the limelight recently as HER3 is an irrefutable partner in HER2-HER3 heterodimer signaling, and HER3 mediates resistance to EGFR- and HER2-targeted therapies [116]. Most *ERBB3* mutations have been identified in the ECD and few in the intracellular KD (Figure 4C). *ERBB3* missense mutations were first reported in 2006, when Jeong et al. found that 1 of 100 colon cancer samples tested had a missense mutation at S846I [117]. However, the authors failed to detect any mutations in 48 lung carcinomas. Ding et al. also identified 3 *ERBB3* somatic mutations (2 missense and 1 non-sense) [118]. Several other studies identified various somatic *ERBB3* mutations in 4% of breast cancer [119], 10% of gastric [120], 1% of ovarian [121, 122], 1% of colon cancer [117], 1% of glioblastoma [123]; 0.5% of squamous carcinomas, and 1% of head and neck cancer [124]. A whole exome sequencing analysis of 72 primary colon tumor specimens by Seshagiri et al. identified *ERBB3* somatic alterations at a rate of 8% (6 out of 72) [125]. Jaiswal et al. performed *ERBB3* exon sequencing of 507 primary tumors [126] and reported *ERBB3* alterations in 1% NSCLC (1/67 squamous; 1/71 adenocarcinoma;), 12% of gastric (11/92), 11% of colon cancers (11/100). Using *in vitro* transformed colonic and breast epithelial model systems, HER3 mutants (V104, A232A, P262H, G284R, T389K, Q809R, S846I and E928G) promoted anchorage-independent growth as compared to WT control in the presence but not in the absence of kinase-active HER2 in a ligand-independent manner [126]. This indicates that mutant HER3 may not be able to induce oncogenic transformation alone but requires HER2 expression to enhance tumor growth, consistent with HER3 being highly kinase-impaired [127] (in the absence of mutations that alter its kinase activity). However, recent studies in our lab indicate that a patient-derived HER3 ECD mutation (T355I) is transforming *in vitro* in the absence of HER2 overexpression. ER+ breast cancer cells (T47D and MCF-7 cells) overexpressing HER3^T355I^ show enhanced colony formation in 3D-Matrigel and enhanced cell proliferation as compared to WT control (manuscript in preparation). The HER3 ECD mutants (V104, A232, P262, G284, D297, G325, and T355I) are common hot-spot mutations across multiple cancers [126] (Figure 4C). Recurrent HER3 KD mutations include S846I and E928G [76]. Umelo et al. identified a novel somatic HER3 kinase mutant (V855A) homologous to EGFR-L858R activating mutation to be a primary driver in lung pathogenesis [128] (Table 2). Using a murine hematopoietic system or transformed human embryonic kidney cells, in the presence of WT ERBB2, ERBB3-V855A demonstrated enhanced transformation of cells upon stimulation with ERBB3’s ligand, NRG1. Figure 4 illustrates the somatic *ERBB3* copy number amplifications (A) and putative activating mutations sequenced from 15198 patients in cBioPortal and GENIE datasets (Figure 4B).

## TARGETED THERAPEUTICS AGAINST HER3 MUTANTS

Multiple agents known to target ERBB receptors directly or indirectly are effective against various ERBB3 mutants. Use of small-molecule inhibitors or antibodies targeting ERBB family members, particularly lapatinib, trastuzumab, and anti-ERBB3 antibodies, were effective in inhibiting mutant ERBB3–mediated oncogenic activity both *in vitro* and *in vivo* [126]. Pertuzumab and afatinib were effective in inhibiting the transforming potential of the HER3^V855A^ mutant [128]. As a part of the SUMMIT trial, neratinib was tested against 16 patients harboring *ERBB3* gene mutations. No clinical activity was observed in *ERBB3* mutant cohort in response to neratinib [105]. One explanation for the lack of clinical activity of neratinib in ERBB3 mutant tumors is the possibility that tumors are not “addicted” to the *ERBB3* mutations. If this is the case, inhibition of HER3 would not block tumor growth. Another prospect is that neratinib, a TKI of EGFR and HER2, may not be effective in *ERBB3*-mutant tumors, where HER3 functions independently of HER2 and an inhibitor specifically targeting HER3 may be necessary. In support of this, shRNA knockdown of *ERBB3 in vivo* in cells with endogenous *ERBB3* mutations moderately but statistically significantly delayed tumor growth [126]. Another potential strategy to treat HER3-mutant tumors may be with antibodies targeting HER3 and other ERBB receptors. For example, Jacobsen et al. generated a mixture of six antibodies (Pan-HER) for synergistic targeting of EGFR, HER2 and HER3, with the goal of preventing compensatory activation of ERBB receptors when only one receptor is inhibited. The authors analyzed the efficacy of Pan-HER2 antibody mixture against 100 different cancer cell lines, including cell lines harboring EGFR or HER2 mutations and/or amplifications, and found that Pan-HER significantly suppressed cancer cell proliferation and outperformed the reference antibodies (cetuximab, transtuzumab and MM-121). Whether Pan-HER blocks the growth of HER3-mutant cancer cells is not known [129].

## HER4 ALTERATIONS

**Figure 5:**
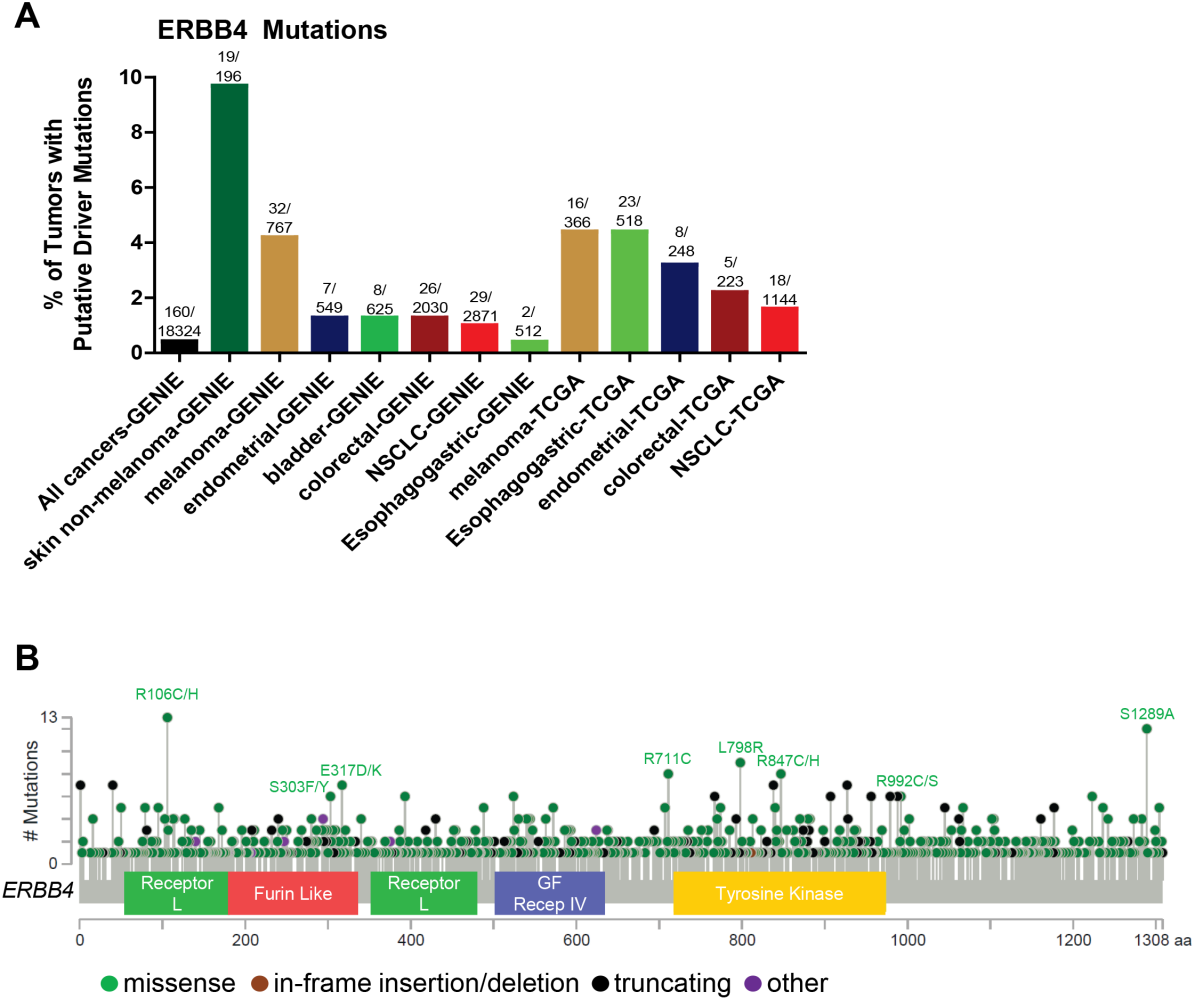
Somatic alterations of *ERBB4* in cancer **(A)** Frequency of *ERBB4* putative driver mutations in selected cBioPortal and GENIE datasets. *ERBB4* copy number amplification is very rare. **(B)** Distribution of somatic variants within *ERBB4* across its domain-annotated protein structure in all cBioPortal studies. NSCLC, non-small cell lung cancer; GBM, GF Recep IV, Growth Factor Receptor IV domain.

Among the members of the ERBB family, *ERBB4* activating mutations and amplifications are the least frequent (Figure 1) and have not been extensively explored. Stephens et al. analyzed 25 breast cancer samples and reported 1 *ERBB4* mutation (4%) outside the KD [130]. Soung et al. screened 595 samples from various cancers including gastric, lung, colon and breast and identified 12 (2%) that contained *ERBB4* KD mutations. Mutations were detected in 1 of 94 breast carcinomas (1.1%), 3 of 104 colorectal carcinomas(2.9%), 5 of 217 non-small cell lung cancers (2.3%) and 3 of 180 gastric carcinomas (1.7%). The authors also analyzed the somatic mutations of *EGFR, ERBB2, PIK3CA, KRAS*, and *BRAF* genes in these 12 samples harboring *ERBB4* mutations and detected a *KRAS* mutation in 1 gastric cancer sample [131]. Prickett et al. screened 79 melanoma patients and identified 24 somatic *ERBB4* mutations in 19% of melanoma patients. Most of these mutations (14) spanned across the extracellular domain (ECD), while three *ERBB4* mutations were identified in the KD. Additionally, there were several mutations which were not associated with any functional domain (P700S, P1033S and R1174Q) and 1 (S1246N) found in His-Me endonuclease domain. The authors also identified that several mutations were multi-mutational hot spots for other oncogenes including NRAS and BRAF. The tumors harboring the *ERBB4* ECD mutants (L39F, R393W, E452K, R491K and R544W) and KD mutant E836K also harbored BRAF mutations. NRAS mutations co-occurred with several *ERBB4* mutants (M3I3I, E317K, E452K, E542K, E563K, P700S R1174Q). The HER4 mutants E317K, E452K, E542K, R544W, E563K, E836K, and E872K induced autophosphorylation as compared to WT control in HEK293T and melanoma cells. HEK293T cells transiently transfected with these HER4 mutants also showed increased *in vitro* kinase activity. Melanoma cells harboring endogenous HER4 mutations showed activation of AKT signaling compared to WT. However, the MAPK pathway was not activated in these cell lines. Knocking down endogenous HER4 using shRNA significantly reduced proliferation of melanoma cells harboring endogenous HER4 mutations as compared to control [132]. Lau et al. described the sequencing techniques to analyze the hot-spot and non-hotspot *ERBB4* gene mutations dispersed across its multiple exons. This technique has been applied within a clinical trial to select patients with *ERBB4*-mutant melanoma for lapatinib treatment [133]. Kurppa et al. functionally characterized 9 HER4 somatic mutations (N181S, T244R, Y285C, R306S, V348L, D595V, H618P, D931Y and K935I) in NSCLC. Out of these, 2 were located in the ECD (Y285C, D595V) and 2 in KD (D931Y and K935I); these mutants were oncogenic and enhanced both basal and NRG1-induced HER4 phosphorylation. All of these HER4 variants also increased activation of endogenous HER2 in the presence of NRG1. The HER4 ECD mutants Y285C and D595V efficiently formed HER4 homodimers when stimulated with NRG1 in NIH 3T3 cells. The above mutants increased phosphorylation and heterodimerization of HER2 in the presence of NRG1 in NIH3T3 cells. The HER4 mutants Y285C, D595C, and K935I promoted prolonged cell survival in NIH 3T3 cells in the absence of serum. These mutants increased HER4 cleavage, resulting in functionally active HER4 soluble intracellular domains (ICDs) as compared to WT, both basally and upon serum starvation in NIH 3T3 cells. This indicates that oncogenic HER4 signaling is transduced through the regulated intra-membrane proteolysis (RIP) pathway rather than canonical RAS-MAPK signaling [134]. This recent identification of HER4 mutations presents a need for a better mechanistic understanding of how HER4 mutations are oncogenic [135]. 160/18324 (0.87%) of all cancers have putative *ERBB4* driver mutations (defined as frequency>5 in cBioPortal or COSMIC databases, or a HotSpot or OncoKB driver annotation in cBioPortal) as per the cBioPortal and GENIE databases (Figure 5). *ERBB4* copy number amplification is very rare. The GENIE database indicates that *ERBB4* driver mutations are most common in skin non-melanomas, but are also found in melanomas, endometrial cancers, bladder cancers, colorectal cancers, NSCLC, and esophagogastric cancers. According to the TCGA, the percentage of tumors with putative *ERBB4* driver mutations are highest in melanoma and esophagogastric, followed by endometrial cancer, colorectal cancer, and NSCLC (Figure 5A). Figure 5B summarizes the distribution of the most common *ERBB4* mutations.

## SENSITIVITY OF HER4 MUTANTS TO DRUGS

The majority of HER4 mutations have been studied in malignant melanoma, where the mutants display a gain-of-function phenotype and were targeted using common EGFR/HER2 tyrosine kinase inhibitors such as lapatinib. Prickett et al. screened several HER4 mutants and targeted the transforming HER4 mutants using the EGFR/HER2 TKI lapatinib. Lapatinib treatment resulted in 10-250 fold inhibition of cell proliferation in cells harboring endogenous HER4 mutants as compared to WT control. Lapatinib inhibited receptor autophosphorylation in a dose-dependent manner. There was also specific inhibition of HER4-induced AKT signaling and markedly enhanced apoptosis in melanoma cells harboring exogenous mutant HER4 as compared to cells harboring WT HER4 [132]. Similarly, Lau et al. highlighted the sensitivity of HER4 mutants to lapatinib. The authors stated that melanoma patients having multiple HER4 mutations showed a wide range of sensitivity to lapatinib and also emphasized the need of comprehensive sequencing strategies for patients harboring two or more HER4 alterations [131]. Unfortunately, no clinical responses to lapatinib were observed in a Phase II trial in HER4-mutant patients [136]. Either a better compound may be needed and/or confirmation of the oncogenic activity of these mutants in additional models is required. Lapatinib is a much less potent inhibitor of HER4 than of EGFR and HER2 [137], and the concentrations needed to inhibit HER4 may not be clinically achievable. Instead, neratinib or afatinib, which are more potent inhibitors of HER4, may be more promising inhibitors of mutant HER4; clinical investigation of these agents in HER4-mutant tumors is warranted.

## CONCLUSION

While inhibitors of EGFR and HER2 have transformed the clinical care of patients harboring alterations in these genes, resistance to EGFR and HER2 inhibitors remains a major problem. Tumor cell plasticity and tumor heterogeneity are major hurdles to prevent acquired resistance to ERBB inhibitors in advanced cancers. The favorable toxicity profile of third-generation EGFR inhibitors such as osimertinib may more easily enable combinations with other agents. Osimertinib is currently in clinical trials in combination with agents including MET inhibitors and immune checkpoint inhibitors, such as PD-L1 inhibitors [138]. Similarly, immune checkpoint blockade is being investigated in combination with HER2 monoclonal antibodies in HER2-amplified breast cancer [139].

Another approach to preventing/delaying drug resistance is to stay one step ahead of the tumor. We are nearing an age in which we can detect new mutations and target them as soon as they are detectable, before tumors have progressed too far. Molecular profiling of drug-resistant tumor tissue or ctDNA following progression on HER family TKIs should become routine in clinical trials in order to identify additional mechanisms of drug resistance. As with anti-HER2 therapies in HER2-amplified breast cancer, moving targeted HER family therapies earlier, to the adjuvant setting, when tumor burden is low and the pool of dividing tumor cells that can acquire new mutations is small, may well result in improved outcomes [69].

More work is needed to systematically characterize the drug sensitivity of each individual ERBB family member mutation. A more detailed understanding of which particular mutations are blocked by which therapies will further enable the promise of precision oncology. In addition, more specific EGFR or HER2 TKIs that spare the considerable toxicity associated with WT EGFR/HER2 inhibition [140, 141] are needed. The HER2-specific TKI tucatinib (ONT-380) does not block EGFR and thus may have more favorable side effects [142]; whether this drug blocks mutant HER2 has not yet been tested. The small molecule EGFR/HER2 TKI AP32788 is more specific for HER2 and EGFR with exon 20 insertions relative to WT receptors [36] and is currently being tested in NSCLC tumors harboring these mutations. Whether AP32788 blocks all known activating HER2 mutants, including non-kinase domain mutants, is not known. Therefore, future drug discovery efforts should focus on agents that selectively block ERBB family member mutants but spare the WT receptors. We are optimistic that the combination of potent, selective inhibitors of ERBB mutants, together with inhibitors of other pathways involved in drug resistance or with immunotherapy, will ultimately lead to durable responses and perhaps cures for the hundreds of thousands of patients with ERBB-driven cancers.

## References

[1] Ullrich A, Coussens L, Hayflick JS, Dull TJ, Gray A, Tam AW, Lee J, Yarden Y, Libermann TA, Schlessinger J, Downward J, Mayes EL, Whittle N (1984). Human epidermal growth factor receptor cDNA sequence and aberrant expression of the amplified gene in A431 epidermoid carcinoma cells. Nature.

[2] Downward J, Yarden Y, Mayes E, Scrace G, Totty N, Stockwell P, Ullrich A, Schlessinger J, Waterfield MD (1984). Close similarity of epidermal growth factor receptor and v-erb-B oncogene protein sequences. Nature.

[3] Schechter AL, Stern DF, Vaidyanathan L, Decker SJ, Drebin JA, Greene MI, Weinberg RA (1984). The neu oncogene: an erb-B-related gene encoding a 185,000-Mr tumour antigen. Nature.

[4] Weiner DB, Liu J, Cohen JA, Williams WV, Greene MI (1989). A point mutation in the neu oncogene mimics ligand induction of receptor aggregation. Nature.

[5] Kraus MH, Issing W, Miki T, Popescu NC, Aaronson SA (1989). Isolation and characterization of ERBB3, a third member of the ERBB/epidermal growth factor receptor family: evidence for overexpression in a subset of human mammary tumors. Proc Natl Acad Sci U S A.

[6] Plowman GD, Whitney GS, Neubauer MG, Green JM, McDonald VL, Todaro GJ, Shoyab M (1990). Molecular cloning and expression of an additional epidermal growth factor receptor-related gene. Proc Natl Acad Sci U S A.

[7] Plowman GD, Culouscou JM, Whitney GS, Green JM, Carlton GW, Foy L, Neubauer MG, Shoyab M (1993). Ligand-specific activation of HER4/p180erbB4, a fourth member of the epidermal growth factor receptor family. Proc Natl Acad Sci U S A.

[8] Garraway LA, Lander ES (2013). Lessons from the cancer genome. Cell.

[9] AACR Project GENIE Consortium (2017). Powering precision medicine through an international consortium. Cancer Discov.

[10] Pao W, Miller V, Zakowski M, Doherty J, Politi K, Sarkaria I, Singh B, Heelan R, Rusch V, Fulton L, Mardis E, Kupfer D, Wilson R (2004). EGF receptor gene mutations are common in lung cancers from “never smokers” and are associated with sensitivity of tumors to gefitinib and erlotinib. Proc Natl Acad Sci U S A.

[11] Lynch TJ, Bell DW, Sordella R, Gurubhagavatula S, Okimoto RA, Brannigan BW, Harris PL, Haserlat SM, Supko JG, Haluska FG, Louis DN, Christiani DC, Settleman J (2004). Activating mutations in the epidermal growth factor receptor underlying responsiveness of non-small-cell lung cancer to gefitinib. N Engl J Med.

[12] Paez JG, Janne PA, Lee JC, Tracy S, Greulich H, Gabriel S, Herman P, Kaye FJ, Lindeman N, Boggon TJ, Naoki K, Sasaki H, Fujii Y (2004). EGFR mutations in lung cancer: correlation with clinical response to gefitinib therapy. Science.

[13] Eberhard DA, Johnson BE, Amler LC, Goddard AD, Heldens SL, Herbst RS, Ince WL, Janne PA, Januario T, Johnson DH, Klein P, Miller VA, Ostland MA (2005). Mutations in the epidermal growth factor receptor and in KRAS are predictive and prognostic indicators in patients with non-small-cell lung cancer treated with chemotherapy alone and in combination with erlotinib. J Clin Oncol.

[14] Kosaka T, Yatabe Y, Endoh H, Kuwano H, Takahashi T, Mitsudomi T (2004). Mutations of the epidermal growth factor receptor gene in lung cancer: biological and clinical implications. Cancer Res.

[15] Yun CH, Boggon TJ, Li Y, Woo MS, Greulich H, Meyerson M, Eck MJ (2007). Structures of lung cancer-derived EGFR mutants and inhibitor complexes: mechanism of activation and insights into differential inhibitor sensitivity. Cancer Cell.

[16] Weinstein IB (2002). Cancer. Addiction to oncogenes--the Achilles heal of cancer. Science.

[17] Chong CR, Janne PA (2013). The quest to overcome resistance to EGFR-targeted therapies in cancer. Nat Med.

[18] Kobayashi S, Boggon TJ, Dayaram T, Janne PA, Kocher O, Meyerson M, Johnson BE, Eck MJ, Tenen DG, Halmos B (2005). EGFR mutation and resistance of non-small-cell lung cancer to gefitinib. N Engl J Med.

[19] Pao W, Miller VA, Politi KA, Riely GJ, Somwar R, Zakowski MF, Kris MG, Varmus H (2005). Acquired resistance of lung adenocarcinomas to gefitinib or erlotinib is associated with a second mutation in the EGFR kinase domain. PLoS Med.

[20] Sharma SV, Bell DW, Settleman J, Haber DA (2007). Epidermal growth factor receptor mutations in lung cancer. Nat Rev Cancer.

[21] Yun CH, Mengwasser KE, Toms AV, Woo MS, Greulich H, Wong KK, Meyerson M, Eck MJ (2008). The T790M mutation in EGFR kinase causes drug resistance by increasing the affinity for ATP. Proc Natl Acad Sci U S A.

[22] Zehir A, Benayed R, Shah RH, Syed A, Middha S, Kim HR, Srinivasan P, Gao J, Chakravarty D, Devlin SM, Hellmann MD, Barron DA, Schram AM (2017). Mutational landscape of metastatic cancer revealed from prospective clinical sequencing of 10,000 patients. Nat Med.

[23] Campbell JD, Alexandrov A, Kim J, Wala J, Berger AH, Pedamallu CS, Shukla SA, Guo G, Brooks AN, Murray BA, Imielinski M, Hu X, Ling S (2016). Distinct patterns of somatic genome alterations in lung adenocarcinomas and squamous cell carcinomas. Nat Genet.

[24] Kwak EL, Jankowski J, Thayer SP, Lauwers GY, Brannigan BW, Harris PL, Okimoto RA, Haserlat SM, Driscoll DR, Ferry D, Muir B, Settleman J, Fuchs CS (2006). Epidermal growth factor receptor kinase domain mutations in esophageal and pancreatic adenocarcinomas. Clin Cancer Res.

[25] Bell DW, Gore I, Okimoto RA, Godin-Heymann N, Sordella R, Mulloy R, Sharma SV, Brannigan BW, Mohapatra G, Settleman J, Haber DA (2005). Inherited susceptibility to lung cancer may be associated with the T790M drug resistance mutation in EGFR. Nat Genet.

[26] Yu HA, Pao W (2013). Targeted therapies: afatinib--new therapy option for EGFR-mutant lung cancer. Nat Rev Clin Oncol.

[27] Campo M, Gerber D, Gainor JF, Heist RS, Temel JS, Shaw AT, Fidias P, Muzikansky A, Engelman JA, Sequist LV (2016). Acquired resistance to first-line afatinib and the challenges of prearranged progression biopsies. J Thorac Oncol.

[28] Mok TS, Wu YL, Ahn MJ, Garassino MC, Kim HR, Ramalingam SS, Shepherd FA, He Y, Akamatsu H, Theelen WS, Lee CK, Sebastian M, Templeton A (2017). Osimertinib or platinum-pemetrexed in EGFR T790M-positive lung cancer. N Engl J Med.

[29] Ramalingam SS, Yang JC, Lee CK, Kurata T, Kim DW, John T, Nogami N, Ohe Y, Mann H, Rukazenkov Y, Ghiorghiu S, Stetson D, Markovets A (2017). Osimertinib as first-line treatment of EGFR mutation-positive advanced non-small-cell lung cancer. J Clin Oncol.

[30] Planchard D, Loriot Y, Andre F, Gobert A, Auger N, Lacroix L, Soria JC (2015). EGFR-independent mechanisms of acquired resistance to AZD9291 in EGFR T790M-positive NSCLC patients. Ann Oncol.

[31] Eberlein CA, Stetson D, Markovets AA, Al-Kadhimi KJ, Lai Z, Fisher PR, Meador CB, Spitzler P, Ichihara E, Ross SJ, Ahdesmaki MJ, Ahmed A, Ratcliffe LE (2015). Acquired resistance to the mutant-selective EGFR inhibitor AZD9291 is associated with increased dependence on RAS signaling in preclinical models. Cancer Res.

[32] Ercan D, Choi HG, Yun CH, Capelletti M, Xie T, Eck MJ, Gray NS, Janne PA (2015). EGFR mutations and resistance to irreversible pyrimidine-based EGFR inhibitors. Clin Cancer Res.

[33] Thress KS, Paweletz CP, Felip E, Cho BC, Stetson D, Dougherty B, Lai Z, Markovets A, Vivancos A, Kuang Y, Ercan D, Matthews SE, Cantarini M (2015). Acquired EGFR C797S mutation mediates resistance to AZD9291 in non-small cell lung cancer harboring EGFR T790M. Nat Med.

[34] Jia Y, Yun CH, Park E, Ercan D, Manuia M, Juarez J, Xu C, Rhee K, Chen T, Zhang H, Palakurthi S, Jang J, Lelais G (2016). Overcoming EGFR(T790M) and EGFR(C797S) resistance with mutant-selective allosteric inhibitors. Nature.

[35] Yang JC, Sequist LV, Geater SL, Tsai CM, Mok TS, Schuler M, Yamamoto N, Yu CJ, Ou SH, Zhou C, Massey D, Zazulina V, Wu YL (2015). Clinical activity of afatinib in patients with advanced non-small-cell lung cancer harbouring uncommon EGFR mutations: a combined post-hoc analysis of LUX-Lung 2, LUX-Lung 3, and LUX-Lung 6. Lancet Oncol.

[36] Gonzalvez F, Zhu X, Huang WS, Baker TE, Ning Y, Wardwell SD, Nadworny S, Zhang S, Das B, Gong Y, Greenfield MT, Jang HG, Kohlmann A (2016). Abstract 2644: AP32788, a potent, selective inhibitor of EGFR and HER2 oncogenic mutants, including exon 20 insertions, in preclinical models. Cancer Res.

[37] Jia Y, Juarez J, Li J, Manuia M, Niederst MJ, Tompkins C, Timple N, Vaillancourt MT, Pferdekamper AC, Lockerman EL, Li C, Anderson J, Costa C (2016). EGF816 exerts anticancer effects in non-small cell lung cancer by irreversibly and selectively targeting primary and acquired activating mutations in the EGF receptor. Cancer Res.

[38] Stupp R, Mason WP, van den Bent MJ, Weller M, Fisher B, Taphoorn MJ, Belanger K, Brandes AA, Marosi C, Bogdahn U, Curschmann J, Janzer RC, Ludwin SK (2005). Radiotherapy plus concomitant and adjuvant temozolomide for glioblastoma. N Engl J Med.

[39] Wong AJ, Ruppert JM, Bigner SH, Grzeschik CH, Humphrey PA, Bigner DS, Vogelstein B (1992). Structural alterations of the epidermal growth factor receptor gene in human gliomas. Proc Natl Acad Sci U S A.

[40] Ekstrand AJ, James CD, Cavenee WK, Seliger B, Pettersson RF, Collins VP (1991). Genes for epidermal growth factor receptor, transforming growth factor alpha, and epidermal growth factor and their expression in human gliomas *in vivo*. Cancer Res.

[41] Aldape KD, Ballman K, Furth A, Buckner JC, Giannini C, Burger PC, Scheithauer BW, Jenkins RB, James CD (2004). Immunohistochemical detection of EGFRvIII in high malignancy grade astrocytomas and evaluation of prognostic significance. J Neuropathol Exp Neurol.

[42] Huang PH, Xu AM, White FM (2009). Oncogenic EGFR signaling networks in glioma. Sci Signal.

[43] Verhaak RG, Hoadley KA, Purdom E, Wang V, Qi Y, Wilkerson MD, Miller CR, Ding L, Golub T, Mesirov JP, Alexe G, Lawrence M, O'Kelly M (2010). Integrated genomic analysis identifies clinically relevant subtypes of glioblastoma characterized by abnormalities in PDGFRA, IDH1, EGFR, and NF1. Cancer Cell.

[44] Brennan CW, Verhaak RG, McKenna A, Campos B, Noushmehr H, Salama SR, Zheng S, Chakravarty D, Sanborn JZ, Berman SH, Beroukhim R, Bernard B, Wu CJ (2013). The somatic genomic landscape of glioblastoma. Cell.

[45] Wen PY, Chang SM, Lamborn KR, Kuhn JG, Norden AD, Cloughesy TF, Robins HI, Lieberman FS, Gilbert MR, Mehta MP, Drappatz J, Groves MD, Santagata S (2014). Phase I/II study of erlotinib and temsirolimus for patients with recurrent malignant gliomas: North American Brain Tumor Consortium trial 04-02. Neuro Oncol.

[46] Raizer JJ, Giglio P, Hu J, Groves M, Merrell R, Conrad C, Phuphanich S, Puduvalli VK, Loghin M, Paleologos N, Yuan Y, Liu D, Rademaker A (2016). A phase II study of bevacizumab and erlotinib after radiation and temozolomide in MGMT unmethylated GBM patients. J Neurooncol.

[47] Uhm JH, Ballman KV, Wu W, Giannini C, Krauss JC, Buckner JC, James CD, Scheithauer BW, Behrens RJ, Flynn PJ, Schaefer PL, Dakhill SR, Jaeckle KA (2011). Phase II evaluation of gefitinib in patients with newly diagnosed Grade 4 astrocytoma: Mayo/North Central Cancer Treatment Group Study N0074. Int J Radiat Oncol Biol Phys.

[48] Chakravarti A, Wang M, Robins HI, Lautenschlaeger T, Curran WJ, Brachman DG, Schultz CJ, Choucair A, Dolled-Filhart M, Christiansen J, Gustavson M, Molinaro A, Mischel P (2013). RTOG 0211: a phase 1/2 study of radiation therapy with concurrent gefitinib for newly diagnosed glioblastoma patients. Int J Radiat Oncol Biol Phys.

[49] Reardon DA, Nabors LB, Mason WP, Perry JR, Shapiro W, Kavan P, Mathieu D, Phuphanich S, Cseh A, Fu Y, Cong J, Wind S, Eisenstat DD (2015). Phase I/randomized phase II study of afatinib, an irreversible ErbB family blocker, with or without protracted temozolomide in adults with recurrent glioblastoma. Neuro Oncol.

[50] Reardon DA, Groves MD, Wen PY, Nabors L, Mikkelsen T, Rosenfeld S, Raizer J, Barriuso J, McLendon RE, Suttle AB, Ma B, Curtis CM, Dar MM (2013). A phase I/II trial of pazopanib in combination with lapatinib in adult patients with relapsed malignant glioma. Clin Cancer Res.

[51] Snuderl M, Fazlollahi L, Le LP, Nitta M, Zhelyazkova BH, Davidson CJ, Akhavanfard S, Cahill DP, Aldape KD, Betensky RA, Louis DN, Iafrate AJ (2011). Mosaic amplification of multiple receptor tyrosine kinase genes in glioblastoma. Cancer Cell.

[52] Szerlip NJ, Pedraza A, Chakravarty D, Azim M, McGuire J, Fang Y, Ozawa T, Holland EC, Huse JT, Jhanwar S, Leversha MA, Mikkelsen T, Brennan CW (2012). Intratumoral heterogeneity of receptor tyrosine kinases EGFR and PDGFRA amplification in glioblastoma defines subpopulations with distinct growth factor response. Proc Natl Acad Sci U S A.

[53] Inda MM, Bonavia R, Mukasa A, Narita Y, Sah DW, Vandenberg S, Brennan C, Johns TG, Bachoo R, Hadwiger P, Tan P, Depinho RA, Cavenee W (2010). Tumor heterogeneity is an active process maintained by a mutant EGFR-induced cytokine circuit in glioblastoma. Genes Dev.

[54] Fan QW, Cheng CK, Gustafson WC, Charron E, Zipper P, Wong RA, Chen J, Lau J, Knobbe-Thomsen C, Weller M, Jura N, Reifenberger G, Shokat KM (2013). EGFR phosphorylates tumor-derived EGFRvIII driving STAT3/5 and progression in glioblastoma. Cancer Cell.

[55] Nathanson DA, Gini B, Mottahedeh J, Visnyei K, Koga T, Gomez G, Eskin A, Hwang K, Wang J, Masui K, Paucar A, Yang H, Ohashi M (2014). Targeted therapy resistance mediated by dynamic regulation of extrachromosomal mutant EGFR DNA. Science.

[56] Westphal M, Maire CL, Lamszus K (2017). EGFR as a target for glioblastoma treatment: an unfulfilled promise. CNS Drugs.

[57] Gan HK, van den Bent M, Lassman AB, Reardon DA, Scott AM (2017). Antibody-drug conjugates in glioblastoma therapy: the right drugs to the right cells. Nat Rev Clin Oncol.

[58] Reilly EB, Phillips AC, Buchanan FG, Kingsbury G, Zhang Y, Meulbroek JA, Cole TB, DeVries PJ, Falls HD, Beam C, Gu J, Digiammarino EL, Palma JP (2015). Characterization of ABT-806, a humanized tumor-specific anti-EGFR monoclonal antibody. Mol Cancer Ther.

[59] Scott AM, Lee FT, Tebbutt N, Herbertson R, Gill SS, Liu Z, Skrinos E, Murone C, Saunder TH, Chappell B, Papenfuss AT, Poon AM, Hopkins W (2007). A phase I clinical trial with monoclonal antibody ch806 targeting transitional state and mutant epidermal growth factor receptors. Proc Natl Acad Sci U S A.

[60] Phillips AC, Boghaert ER, Vaidya KS, Mitten MJ, Norvell S, Falls HD, DeVries PJ, Cheng D, Meulbroek JA, Buchanan FG, McKay LM, Goodwin NC, Reilly EB (2016). ABT-414, an antibody-drug conjugate targeting a tumor-selective EGFR epitope. Mol Cancer Ther.

[61] Reardon DA, Lassman AB, van den Bent M, Kumthekar P, Merrell R, Scott AM, Fichtel L, Sulman EP, Gomez E, Fischer J, Lee HJ, Munasinghe W, Xiong H (2017). Efficacy and safety results of ABT-414 in combination with radiation and temozolomide in newly diagnosed glioblastoma. Neuro Oncol.

[62] Chistiakov DA, Chekhonin IV, Chekhonin VP (2017). The EGFR variant III mutant as a target for immunotherapy of glioblastoma multiforme. Eur J Pharmacol.

[63] Del Vecchio CA, Wong AJ (2010). Rindopepimut, a 14-mer injectable peptide vaccine against EGFRvIII for the potential treatment of glioblastoma multiforme. Curr Opin Mol Ther.

[64] Heimberger AB, Archer GE, Crotty LE, McLendon RE, Friedman AH, Friedman HS, Bigner DD, Sampson JH (2002). Dendritic cells pulsed with a tumor-specific peptide induce long-lasting immunity and are effective against murine intracerebral melanoma. Neurosurgery.

[65] Weller M, Butowski N, Tran DD, Recht LD, Lim M, Hirte H, Ashby L, Mechtler L, Goldlust SA, Iwamoto F, Drappatz J, O'Rourke DM, Wong M (2017). Rindopepimut with temozolomide for patients with newly diagnosed, EGFRvIII-expressing glioblastoma (ACT IV): a randomised, double-blind, international phase 3 trial. Lancet Oncol.

[66] Slamon DJ, Clark GM, Wong SG, Levin WJ, Ullrich A, McGuire WL (1987). Human breast cancer: correlation of relapse and survival with amplification of the HER-2/neu oncogene. Science.

[67] Gravalos C, Jimeno A (2008). HER2 in gastric cancer: a new prognostic factor and a novel therapeutic target. Ann Oncol.

[68] Jimenez RE, Hussain M, Bianco FJ, Vaishampayan U, Tabazcka P, Sakr WA, Pontes JE, Wood DP, Grignon DJ (2001). Her-2/neu overexpression in muscle-invasive urothelial carcinoma of the bladder: prognostic significance and comparative analysis in primary and metastatic tumors. Clin Cancer Res.

[69] Arteaga CL, Engelman JA (2014). ERBB receptors: from oncogene discovery to basic science to mechanism-based cancer therapeutics. Cancer Cell.

[70] Samson P, Lockhart AC (2017). Biologic therapy in esophageal and gastric malignancies: current therapies and future directions. J Gastrointest Oncol.

[71] Stephens P, Hunter C, Bignell G, Edkins S, Davies H, Teague J, Stevens C, O'Meara S, Smith R, Parker A, Barthorpe A, Blow M, Brackenbury L (2004). Lung cancer: intragenic ERBB2 kinase mutations in tumours. Nature.

[72] Shigematsu H, Takahashi T, Nomura M, Majmudar K, Suzuki M, Lee H, Wistuba II, Fong KM, Toyooka S, Shimizu N, Fujisawa T, Minna JD, Gazdar AF (2005). Somatic mutations of the HER2 kinase domain in lung adenocarcinomas. Cancer Res.

[73] Wang SE, Narasanna A, Perez-Torres M, Xiang B, Wu FY, Yang S, Carpenter G, Gazdar AF, Muthuswamy SK, Arteaga CL (2006). HER2 kinase domain mutation results in constitutive phosphorylation and activation of HER2 and EGFR and resistance to EGFR tyrosine kinase inhibitors. Cancer Cell.

[74] Lee JW, Soung YH, Seo SH, Kim SY, Park CH, Wang YP, Park K, Nam SW, Park WS, Kim SH, Lee JY, Yoo NJ, Lee SH (2006). Somatic mutations of ERBB2 kinase domain in gastric, colorectal, and breast carcinomas. Clin Cancer Res.

[75] Chmielecki J, Ross JS, Wang K, Frampton GM, Palmer GA, Ali SM, Palma N, Morosini D, Miller VA, Yelensky R, Lipson D, Stephens PJ (2015). Oncogenic alterations in ERBB2/HER2 represent potential therapeutic targets across tumors from diverse anatomic sites of origin. Oncologist.

[76] Cancer Genome Atlas Network (2012). Comprehensive molecular portraits of human breast tumours. Nature.

[77] Bose R, Kavuri SM, Searleman AC, Shen W, Shen D, Koboldt DC, Monsey J, Goel N, Aronson AB, Li S, Ma CX, Ding L, Mardis ER (2013). Activating HER2 mutations in HER2 gene amplification negative breast cancer. Cancer Discov.

[78] Ross JS, Gay LM, Wang K, Ali SM, Chumsri S, Elvin JA, Bose R, Vergilio JA, Suh J, Yelensky R, Lipson D, Chmielecki J, Waintraub S (2016). Nonamplification ERBB2 genomic alterations in 5605 cases of recurrent and metastatic breast cancer: an emerging opportunity for anti-HER2 targeted therapies. Cancer.

[79] Kavuri SM, Jain N, Galimi F, Cottino F, Leto SM, Migliardi G, Searleman AC, Shen W, Monsey J, Trusolino L, Jacobs SA, Bertotti A, Bose R (2015). HER2 activating mutations are targets for colorectal cancer treatment. Cancer Discov.

[80] de Martino M, Zhuang D, Klatte T, Rieken M, Roupret M, Xylinas E, Clozel T, Krzywinski M, Elemento O, Shariat SF (2014). Impact of ERBB2 mutations on *in vitro* sensitivity of bladder cancer to lapatinib. Cancer Biol Ther.

[81] Wang T, Xu Y, Sheng S, Yuan H, Ouyang T, Li J, Wang T, Fan Z, Fan T, Lin B, Xie Y (2017). HER2 somatic mutations are associated with poor survival in HER2-negative breast cancers. Cancer Sci.

[82] Shimamura T, Ji H, Minami Y, Thomas RK, Lowell AM, Shah K, Greulich H, Glatt KA, Meyerson M, Shapiro GI, Wong KK (2006). Non-small-cell lung cancer and Ba/F3 transformed cells harboring the ERBB2 G776insV_G/C mutation are sensitive to the dual-specific epidermal growth factor receptor and ERBB2 inhibitor HKI-272. Cancer Res.

[83] Gazdar AF, Shigematsu H, Herz J, Minna JD (2004). Mutations and addiction to EGFR: the Achilles 'heal' of lung cancers?. Trends Mol Med.

[84] Perera SA, Li D, Shimamura T, Raso MG, Ji H, Chen L, Borgman CL, Zaghlul S, Brandstetter KA, Kubo S, Takahashi M, Chirieac LR, Padera RF (2009). HER2YVMA drives rapid development of adenosquamous lung tumors in mice that are sensitive to BIBW2992 and rapamycin combination therapy. Proc Natl Acad Sci U S A.

[85] Kancha RK, von Bubnoff N, Bartosch N, Peschel C, Engh RA, Duyster J (2011). Differential sensitivity of ERBB2 kinase domain mutations towards lapatinib. PLoS One.

[86] Asahina H, Yamazaki K, Kinoshita I, Yokouchi H, Dosaka-Akita H, Nishimura M (2006). Non-responsiveness to gefitinib in a patient with lung adenocarcinoma having rare EGFR mutations S768I and V769L. Lung Cancer.

[87] Chen Y, Takita J, Choi YL, Kato M, Ohira M, Sanada M, Wang L, Soda M, Kikuchi A, Igarashi T, Nakagawara A, Hayashi Y, Mano H (2008). Oncogenic mutations of ALK kinase in neuroblastoma. Nature.

[88] George RE, Sanda T, Hanna M, Frohling S, Luther W, Zhang J, Ahn Y, Zhou W, London WB, McGrady P, Xue L, Zozulya S, Gregor VE (2008). Activating mutations in ALK provide a therapeutic target in neuroblastoma. Nature.

[89] Janoueix-Lerosey I, Lequin D, Brugieres L, Ribeiro A, de Pontual L, Combaret V, Raynal V, Puisieux A, Schleiermacher G, Pierron G, Valteau-Couanet D, Frebourg T, Michon J (2008). Somatic and germline activating mutations of the ALK kinase receptor in neuroblastoma. Nature.

[90] Greulich H, Kaplan B, Mertins P, Chen TH, Tanaka KE, Yun CH, Zhang X, Lee SH, Cho J, Ambrogio L, Liao R, Imielinski M, Banerji S (2012). Functional analysis of receptor tyrosine kinase mutations in lung cancer identifies oncogenic extracellular domain mutations of ERBB2. Proc Natl Acad Sci U S A.

[91] Hanker AB, Brewer MR, Sheehan JH, Koch JP, Sliwoski GR, Nagy R, Lanman R, Berger MF, Hyman DM, Solit DB, He J, Miller V, Cutler RE (2017). An acquired HER2T798I gatekeeper mutation induces resistance to neratinib in a patient with HER2 mutant-driven breast cancer. Cancer Discov.

[92] Zabransky DJ, Yankaskas CL, Cochran RL, Wong HY, Croessmann S, Chu D, Kavuri SM, Red Brewer M, Rosen DM, Dalton WB, Cimino-Mathews A, Cravero K, Button B (2015). HER2 missense mutations have distinct effects on oncogenic signaling and migration. Proc Natl Acad Sci U S A.

[93] Minami Y, Shimamura T, Shah K, LaFramboise T, Glatt KA, Liniker E, Borgman CL, Haringsma HJ, Feng W, Weir BA, Lowell AM, Lee JC, Wolf J (2007). The major lung cancer-derived mutants of ERBB2 are oncogenic and are associated with sensitivity to the irreversible EGFR/ERBB2 inhibitor HKI-272. Oncogene.

[94] Ali SM, Alpaugh RK, Downing SR, Stephens PJ, Yu JQ, Wu H, Buell JK, Miller VA, Lipson D, Palmer GA, Ross JS, Cristofanilli M (2014). Response of an ERBB2-mutated inflammatory breast carcinoma to human epidermal growth factor receptor 2-targeted therapy. J Clin Oncol.

[95] Chumsri S, Weidler J, Ali S, Balasubramanian S, Wallweber G, DeFazio-Eli L, Chenna A, Huang W, DeRidder A, Goicocheal L, Perez EA (2015). Prolonged response to trastuzumab in a patient with HER2-nonamplified breast cancer with elevated HER2 dimerization harboring an ERBB2 S310F mutation. J Natl Compr Canc Netw.

[96] Chuang JC, Stehr H, Liang Y, Das M, Huang J, Diehn M, Wakelee HA, Neal JW (2017). ERBB2-mutated metastatic non-small cell lung cancer: response and resistance to targeted therapies. J Thorac Oncol.

[97] Mazieres J, Barlesi F, Filleron T, Besse B, Monnet I, Beau-Faller M, Peters S, Dansin E, Fruh M, Pless M, Rosell R, Wislez M, Fournel P (2016). Lung cancer patients with HER2 mutations treated with chemotherapy and HER2-targeted drugs: results from the European EUHER2 cohort. Ann Oncol.

[98] Ben-Baruch NE, Bose R, Kavuri SM, Ma CX, Ellis MJ (2015). HER2-mutated breast cancer responds to treatment with single-agent neratinib, a second-generation HER2/EGFR tyrosine kinase inhibitor. J Natl Compr Canc Netw.

[99] Gandhi L, Bahleda R, Tolaney SM, Kwak EL, Cleary JM, Pandya SS, Hollebecque A, Abbas R, Ananthakrishnan R, Berkenblit A, Krygowski M, Liang Y, Turnbull KW (2014). Phase I study of neratinib in combination with temsirolimus in patients with human epidermal growth factor receptor 2-dependent and other solid tumors. J Clin Oncol.

[100] De Greve J, Teugels E, Geers C, Decoster L, Galdermans D, De Mey J, Everaert H, Umelo I, In't Veld P, Schallier D (2012). Clinical activity of afatinib (BIBW 2992) in patients with lung adenocarcinoma with mutations in the kinase domain of HER2/neu. Lung Cancer.

[101] Costa DB, Jorge SE, Moran JP, Freed JA, Zerillo JA, Huberman MS, Kobayashi SS (2016). Pulse afatinib for ERBB2 Exon 20 insertion-mutated lung adenocarcinomas. J Thorac Oncol.

[102] Kris MG, Camidge DR, Giaccone G, Hida T, Li BT, O'Connell J, Taylor I, Zhang H, Arcila ME, Goldberg Z, Janne PA (2015). Targeting HER2 aberrations as actionable drivers in lung cancers: phase II trial of the pan-HER tyrosine kinase inhibitor dacomitinib in patients with HER2-mutant or amplified tumors. Ann Oncol.

[103] Ma CX, Bose R, Gao F, Freedman RA, Telli ML, Kimmick G, Winer E, Naughton M, Goetz MP, Russell C, Tripathy D, Cobleigh M, Forero A (2017). Neratinib efficacy and circulating tumor DNA detection of HER2 mutations in HER2 nonamplified metastatic breast cancer. Clin Cancer Res.

[104] Hyman D, Piha-Paul S, Saura C, Arteaga C, Mayer I, Shapiro G, Loi S, Lalani A, Xu F, Cutler R, Butturini A, Bryce R, Meric-Bernstam F (2017). Abstract PD2–08: neratinib + fulvestrant in ERBB2-mutant, HER2–non-amplified, estrogen receptor (ER)-positive, metastatic breast cancer (MBC): preliminary analysis from the phase II SUMMIT trial. Cancer Res.

[105] Hyman DM, Piha-Paul SA, Rodon J, Saura C, Shapiro GI, Quinn DI, Moreno V, Mayer IA, Arteaga C, Boni V, Calvo E, Loi S, Lockhart AC (2017). Abstract CT001: neratinib in HER2 or HER3 mutant solid tumors: SUMMIT, a global, multi-histology, open-label, phase 2 “basket” study. Cancer Res.

[106] Kosaka T, Tanizaki J, Paranal RM, Endoh H, Lydon C, Capelletti M, Repellin CE, Choi J, Ogino A, Calles A, Ercan D, Redig AJ, Bahcall M (2017). Response heterogeneity of EGFR and HER2 Exon 20 insertions to covalent EGFR and HER2 inhibitors. Cancer Res.

[107] Carmona FJ, Hyman D, Ulaner G, Erinjeri J, Bouvier N, Won H, Cutler R, Alani A, Berger M, Baselga J, Scaltriti M (2016). Abstract 298: amplification of mutant ERBB2 drives resistance to the irreversible kinase inhibitor neratinib in ERBB2-mutated breast cancer patients. Cancer Res.

[108] Wen W, Chen WS, Xiao N, Bender R, Ghazalpour A, Tan Z, Swensen J, Millis SZ, Basu G, Gatalica Z, Press MF (2015). Mutations in the kinase domain of the HER2/ERBB2 gene identified in a wide variety of human cancers. J Mol Diagn.

[109] Boulbes DR, Arold ST, Chauhan GB, Blachno KV, Deng N, Chang WC, Jin Q, Huang TH, Hsu JM, Brady SW, Bartholomeusz C, Ladbury JE, Stone S (2015). HER family kinase domain mutations promote tumor progression and can predict response to treatment in human breast cancer. Mol Oncol.

[110] Zuo WJ, Jiang YZ, Wang YJ, Xu XE, Hu X, Liu GY, Wu J, Di GH, Yu KD, Shao ZM (2016). Dual characteristics of novel HER2 kinase domain mutations in response to HER2-targeted therapies in human breast cancer. Clin Cancer Res.

[111] Rexer BN, Ghosh R, Narasanna A, Estrada MV, Chakrabarty A, Song Y, Engelman JA, Arteaga CL (2013). Human breast cancer cells harboring a gatekeeper T798M mutation in HER2 overexpress EGFR ligands and are sensitive to dual inhibition of EGFR and HER2. Clin Cancer Res.

[112] Xu X, De Angelis C, Burke KA, Nardone A, Hu H, Qin L, Veeraraghavan J, Sethunath V, Heiser LM, Wang N, Ng CK, Chen ES, Renwick A (2017). HER2 reactivation through acquisition of the HER2 L755S mutation as a mechanism of acquired resistance to HER2-targeted therapy in HER2+ breast cancer. Clin Cancer Res.

[113] Takezawa K, Pirazzoli V, Arcila ME, Nebhan CA, Song X, de Stanchina E, Ohashi K, Janjigian YY, Spitzler PJ, Melnick MA, Riely GJ, Kris MG, Miller VA (2012). HER2 amplification: a potential mechanism of acquired resistance to EGFR inhibition in EGFR-mutant lung cancers that lack the second-site EGFRT790M mutation. Cancer Discov.

[114] Yu HA, Arcila ME, Rekhtman N, Sima CS, Zakowski MF, Pao W, Kris MG, Miller VA, Ladanyi M, Riely GJ (2013). Analysis of tumor specimens at the time of acquired resistance to EGFR-TKI therapy in 155 patients with EGFR-mutant lung cancers. Clin Cancer Res.

[115] Arcila ME, Chaft JE, Nafa K, Roy-Chowdhuri S, Lau C, Zaidinski M, Paik PK, Zakowski MF, Kris MG, Ladanyi M (2012). Prevalence, clinicopathologic associations, and molecular spectrum of ERBB2 (HER2) tyrosine kinase mutations in lung adenocarcinomas. Clin Cancer Res.

[116] Sergina NV, Rausch M, Wang D, Blair J, Hann B, Shokat KM, Moasser MM (2007). Escape from HER-family tyrosine kinase inhibitor therapy by the kinase-inactive HER3. Nature.

[117] Jeong EG, Soung YH, Lee JW, Lee SH, Nam SW, Lee JY, Yoo NJ, Lee SH (2006). ERBB3 kinase domain mutations are rare in lung, breast and colon carcinomas. Int J Cancer.

[118] Ding L, Getz G, Wheeler DA, Mardis ER, McLellan MD, Cibulskis K, Sougnez C, Greulich H, Muzny DM, Morgan MB, Fulton L, Fulton RS, Zhang Q (2008). Somatic mutations affect key pathways in lung adenocarcinoma. Nature.

[119] Kan Z, Jaiswal BS, Stinson J, Janakiraman V, Bhatt D, Stern HM, Yue P, Haverty PM, Bourgon R, Zheng J, Moorhead M, Chaudhuri S, Tomsho LP (2010). Diverse somatic mutation patterns and pathway alterations in human cancers. Nature.

[120] Wang K, Kan J, Yuen ST, Shi ST, Chu KM, Law S, Chan TL, Kan Z, Chan AS, Tsui WY, Lee SP, Ho SL, Chan AK (2011). Exome sequencing identifies frequent mutation of ARID1A in molecular subtypes of gastric cancer. Nat Genet.

[121] Greenman C, Stephens P, Smith R, Dalgliesh GL, Hunter C, Bignell G, Davies H, Teague J, Butler A, Stevens C, Edkins S, O'Meara S, Vastrik I (2007). Patterns of somatic mutation in human cancer genomes. Nature.

[122] Cancer Genome Atlas Research Network (2011). Integrated genomic analyses of ovarian carcinoma. Nature.

[123] Cancer Genome Atlas Research Network (2008). Comprehensive genomic characterization defines human glioblastoma genes and core pathways. Nature.

[124] Stransky N, Egloff AM, Tward AD, Kostic AD, Cibulskis K, Sivachenko A, Kryukov GV, Lawrence MS, Sougnez C, McKenna A, Shefler E, Ramos AH, Stojanov P (2011). The mutational landscape of head and neck squamous cell carcinoma. Science.

[125] Seshagiri S, Stawiski EW, Durinck S, Modrusan Z, Storm EE, Conboy CB, Chaudhuri S, Guan Y, Janakiraman V, Jaiswal BS, Guillory J, Ha C, Dijkgraaf GJ (2012). Recurrent R-spondin fusions in colon cancer. Nature.

[126] Jaiswal BS, Kljavin NM, Stawiski EW, Chan E, Parikh C, Durinck S, Chaudhuri S, Pujara K, Guillory J, Edgar KA, Janakiraman V, Scholz RP, Bowman KK (2013). Oncogenic ERBB3 mutations in human cancers. Cancer Cell.

[127] Shi F, Telesco SE, Liu Y, Radhakrishnan R, Lemmon MA (2010). ErbB3/HER3 intracellular domain is competent to bind ATP and catalyze autophosphorylation. Proc Natl Acad Sci U S A.

[128] Umelo I, Noeparast A, Chen G, Renard M, Geers C, Vansteenkiste J, Giron P, De Wever O, Teugels E, De Greve J (2016). Identification of a novel HER3 activating mutation homologous to EGFR-L858R in lung cancer. Oncotarget.

[129] Jacobsen HJ, Poulsen TT, Dahlman A, Kjaer I, Koefoed K, Sen JW, Weilguny D, Bjerregaard B, Andersen CR, Horak ID, Pedersen MW, Kragh M, Lantto J (2015). Pan-HER, an antibody mixture simultaneously targeting EGFR, HER2, and HER3, effectively overcomes tumor heterogeneity and plasticity. Clin Cancer Res.

[130] Stephens P, Edkins S, Davies H, Greenman C, Cox C, Hunter C, Bignell G, Teague J, Smith R, Stevens C, O'Meara S, Parker A, Tarpey P (2005). A screen of the complete protein kinase gene family identifies diverse patterns of somatic mutations in human breast cancer. Nat Genet.

[131] Soung YH, Lee JW, Kim SY, Wang YP, Jo KH, Moon SW, Park WS, Nam SW, Lee JY, Yoo NJ, Lee SH (2006). Somatic mutations of the ERBB4 kinase domain in human cancers. Int J Cancer.

[132] Prickett TD, Agrawal NS, Wei X, Yates KE, Lin JC, Wunderlich JR, Cronin JC, Cruz P, Rosenberg SA, Samuels Y (2009). Analysis of the tyrosine kinome in melanoma reveals recurrent mutations in ERBB4. Nat Genet.

[133] Lau C, Killian KJ, Samuels Y, Rudloff U (2014). ERBB4 mutation analysis: emerging molecular target for melanoma treatment. Methods Mol Biol.

[134] Kurppa KJ, Denessiouk K, Johnson MS, Elenius K (2016). Activating ERBB4 mutations in non-small cell lung cancer. Oncogene.

[135] Rudloff U, Samuels Y (2010). A growing family: adding mutated Erbb4 as a novel cancer target. Cell Cycle.

[136] Gonzalez-Cao M, Rodon J, Karachaliou N, Sanchez J, Santarpia M, Viteri S, Pilotto S, Teixido C, Riso A, Rosell R (2015). Other targeted drugs in melanoma. Ann Transl Med.

[137] Rusnak DW, Lackey K, Affleck K, Wood ER, Alligood KJ, Rhodes N, Keith BR, Murray DM, Knight WB, Mullin RJ, Gilmer TM (2001). The effects of the novel, reversible epidermal growth factor receptor/ErbB-2 tyrosine kinase inhibitor, GW2016, on the growth of human normal and tumor-derived cell lines *in vitro* and *in vivo*. Mol Cancer Ther.

[138] Russo A, Franchina T, Ricciardi GR, Smiroldo V, Picciotto M, Zanghi M, Rolfo C, Adamo V (2017). Third generation EGFR TKIs in EGFR-mutated NSCLC: where are we now and where are we going. Crit Rev Oncol Hematol.

[139] Pusztai L, Karn T, Safonov A, Abu-Khalaf MM, Bianchini G (2016). New strategies in breast cancer: immunotherapy. Clin Cancer Res.

[140] Lucchini E, Pilotto S, Spada E, Melisi D, Bria E, Tortora G (2014). Targeting the epidermal growth factor receptor in solid tumors: focus on safety. Expert Opin Drug Saf.

[141] Chan A (2016). Neratinib in HER-2-positive breast cancer: results to date and clinical usefulness. Ther Adv Med Oncol.

[142] Moulder SL, Borges VF, Baetz T, McSpadden T, Fernetich G, Murthy RK, Chavira R, Guthrie K, Barrett E, Chia SK (2017). Phase I study of ONT-380, a HER2 inhibitor, in patients with HER2+-advanced solid tumors, with an expansion cohort in HER2+ metastatic breast cancer (MBC). Clin Cancer Res.

[143] Lee JC, Vivanco I, Beroukhim R, Huang JH, Feng WL, DeBiasi RM, Yoshimoto K, King JC, Nghiemphu P, Yuza Y, Xu Q, Greulich H, Thomas RK (2006). Epidermal growth factor receptor activation in glioblastoma through novel missense mutations in the extracellular domain. PLoS Med.

[144] Kobayashi Y, Togashi Y, Yatabe Y, Mizuuchi H, Jangchul P, Kondo C, Shimoji M, Sato K, Suda K, Tomizawa K, Takemoto T, Hida T, Nishio K (2015). EGFR Exon 18 mutations in lung cancer: molecular predictors of augmented sensitivity to afatinib or neratinib as compared with first- or third-generation TKIs. Clin Cancer Res.

[145] Wu JY, Yu CJ, Chang YC, Yang CH, Shih JY, Yang PC (2011). Effectiveness of tyrosine kinase inhibitors on “uncommon” epidermal growth factor receptor mutations of unknown clinical significance in non-small cell lung cancer. Clin Cancer Res.

[146] He M, Capelletti M, Nafa K, Yun CH, Arcila ME, Miller VA, Ginsberg MS, Zhao B, Kris MG, Eck MJ, Janne PA, Ladanyi M, Oxnard GR (2012). EGFR exon 19 insertions: a new family of sensitizing EGFR mutations in lung adenocarcinoma. Clin Cancer Res.

[147] Yasuda H, Park E, Yun CH, Sng NJ, Lucena-Araujo AR, Yeo WL, Huberman MS, Cohen DW, Nakayama S, Ishioka K, Yamaguchi N, Hanna M, Oxnard GR (2013). Structural, biochemical, and clinical characterization of epidermal growth factor receptor (EGFR) exon 20 insertion mutations in lung cancer. Sci Transl Med.

[148] Yasuda H, Kobayashi S, Costa DB (2012). EGFR exon 20 insertion mutations in non-small-cell lung cancer: preclinical data and clinical implications. Lancet Oncol.

[149] Chiu CH, Yang CT, Shih JY, Huang MS, Su WC, Lai RS, Wang CC, Hsiao SH, Lin YC, Ho CL, Hsia TC, Wu MF, Lai CL (2015). Epidermal growth factor receptor tyrosine kinase inhibitor treatment response in advanced lung adenocarcinomas with G719X/L861Q/S768I mutations. J Thorac Oncol.

[150] Mazieres J, Peters S, Lepage B, Cortot AB, Barlesi F, Beau-Faller M, Besse B, Blons H, Mansuet-Lupo A, Urban T, Moro-Sibilot D, Dansin E, Chouaid C (2013). Lung cancer that harbors an HER2 mutation: epidemiologic characteristics and therapeutic perspectives. J Clin Oncol.

